# Microbial Biofilms Dynamics and Functionality in an Urban *Mycobacterium*-Dominated Drinking Water Distribution System

**DOI:** 10.1021/acs.est.5c09194

**Published:** 2026-02-09

**Authors:** Valentin Gangloff, Borja Aldeguer-Riquelme, M. Adela Yañez, Gabrielle Potocki-Veronese, Etienne Severac, Josefa Antón, Elena Soria, Fernando Santos

**Affiliations:** † Department of Physiology, Genetics, and Microbiology, University of Alicante, Alicante 03080, Spain; ‡ School of Civil & Environmental Engineering and School of Biological Sciences, 1372 Georgia Institute of Technology, Atlanta, Georgia 30332, United States; § LABAQUA, S.A.U. Alicante 03114, Spain; ∥ TBI, Université de Toulouse, CNRS, INRAE, INSA, Toulouse 31077, France; ⊥ Multidisciplinary Institute of Environmental Studies Ramon Margalef, University of Alicante, Alicante 03080, Spain; # Applied Biomedicine Group (Alicante Institute for Health and Biomedical Research, ISABIAL), Alicante 03080, Spain

**Keywords:** DWDS, biofilm, Mycobacterium, chlorination, diversity, MAGs

## Abstract

Microbial communities in drinking water distribution systems (DWDS) develop primarily as biofilms on pipe surfaces. Despite their impact on water quality, infrastructure maintenance, and biosafety, biofilms are not routinely controlled. In this study, we investigated the bacterial community dynamics and functionality in an urban chlorinated DWDS, dominated by *Mycobacterium*, through a multiphasic approach which included 16S rRNA gene metabarcoding, metagenomics and microscopy. Our results showed that biofilm communities were more functionally diverse compared to those from water and that the biofilm maturity was positively correlated with the prevalence of potential *Mycobacterium* emerging pathogens and a broader distribution of antibiotic resistance genes (ARGs) within the microbial community. The reconstruction of metagenome-assembled genomes (MAGs) and the corresponding genomospecies allowed the identification of key microbial taxa involved in the biofilm matrix remodeling, with 22% of them strongly responsible for biofilm formation. A diverse and novel viral community was detected across the system, including new putative *Mycobacterium* phages that might act against mycolic acids and thus contribute to biofilm destabilization. Our findings enhance our understanding of DWDS microbial composition and biofilm formation dynamics, focusing on “who does what” and then providing a foundation for developing effective biofilm control strategies in water distribution systems.

## Graphical abstract

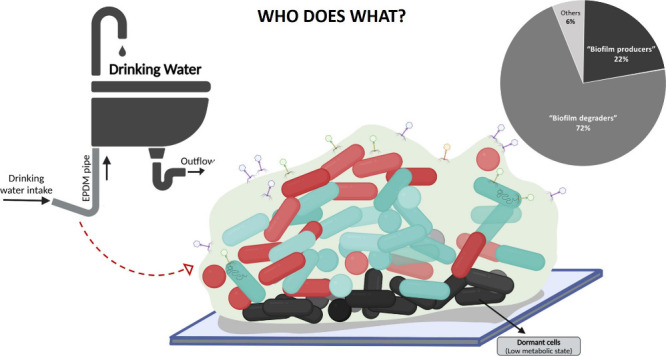

## Introduction

Microbes are present in drinking water distribution systems (DWDS) as part of the autochthonous water microbiota or because they infiltrate as contaminants due to infrastructural deficiencies. While most microbes in DWDS are harmless, those considered pathogenic might pose significant health risks if they are not adequately controlled. Therefore, maintenance of the water quality, infrastructure integrity, and biosafety are priorities in water control programs. Advances in microbiology research have facilitated the monitoring of microbial communities in DWDS, providing a deeper understanding of how the composition of the drinking water microbiome is influenced by the source of the water, the disinfection treatment employed and the characteristics of the pipe material, which seems to be the principal factor determining the community composition.^1–3^

Although drinking water typically contains planktonic bacterial numbers between 10^3^ and 10^6^ cells/mL,^4^ up to 90% of the total bacterial biomass in DWDS is found in the biofilms that develop in the interfaces between the water and pipe walls, even in the presence of disinfectants or reduced amounts of organic carbon.^5^ The presence of biofilms in DWDS is a well-known primary cause of water quality problems and the biofilm detachment of cells into the bulk water is a major biosafety concern if released bacteria are pathogenic and/or antibiotic resistant.^6,7^ Biofilms also participate in pipe corrosion, weakening metal structures, producing undesirable tastes and smells to drinking water, and facilitating the entry of external contaminants into the system. Finally, biofilm formation and growth can lead to pipe obstruction and reduced efficiency of the water treatment processes. This biofouling hinders water flow and requires frequent cleaning or even replacement of system components, resulting in increased costs for water utilities.^8^

However, in spite of the biofilm impact, regulations of water quality and safety of urban DWDS do not consider the control of biofilms and focus solely on determinations of fecal microbial markers, pathogenic bacteria such as *Legionella* or *Clostridium*, and total heterotrophic bacteria plate counts as the regular parameters.^9^ Additionally, scientific studies analyzing biofilms from real urban networks are scarce^10^ and, to our knowledge, none of them have employed state-of-the-art metagenomic analyses with the aim of identifying which members of the DWDS microbial communities can be associated to the formation, evolution and persistence of the biofilm. Such information is essential to develop safe and effective treatments to prevent or disrupt biofilms. Indeed, European Regulation EU 2020/2184 requires the implementation of measures that directly impact the formation and control of biofilms. This issue should be addressed within the Water Safety Plans of drinking water distribution systems.

In this work, an in-depth multiphasic approach, including 16S rRNA gene amplicon sequencing (metabarcoding), metagenomics, CARD-fluorescence *in situ* hybridization and analytical chemistry, has been applied to study the dynamics and functional aspects of the microbial communities in an urban *Mycobacterium*-dominated DWDS, including virus-host interactions. Our results show how unconnected areas of the same DWDS harbor similar microbial communities and to what extent the degree of biofilm maturity is intimately tied to an increasing abundance of potential pathogens and ARGs, which might constitute targets for the development of molecular diagnostic tools for rapid decision making on water sustainability plans. Statistical analyses allowed distinguishing which bacteria were “specific” or “prevalent” in biofilms or in the planktonic fraction. In addition, we provide insights into relevant functions linked to different biofilm stages based on the reconstruction of metagenome-assembled genomes (MAGs). Thus, this study advances our understanding of the microbial dynamics and interactions in DWDS biofilms, which has implications on water quality and network management by establishing modern water safety plans.

## Materials and Methods

### Sampling and Physicochemical Determinations of Water Samples

Twelve biofilms and water samples from six buildings, A–F (Figure 1A), included in an urban DWDS in the Southeast of Spain and supplied with the same chlorinated mixed water (freshwater from aquifers mixed with desalinated seawater) were obtained in May 2021. Biofilm biomass, developed on ethylene propylene diene monomer (EPDM) hoses that connected the DWDS to individual washbasins (Figure 1B), was collected with a sterile spatula and kept at −80 °C (Table S1). While the time in place for devices A-F was unknown, an additional hose G was sampled one year after its installation. In addition, 50 L of water from each sampling point were filtered through 0.22-μm pore-size polyvinylidene fluoride membranes (Millipore) and filters kept at −80 °C. A physicochemical analysis was performed with the water samples by the company LABAQUA, S.A.U. accredited under ISO 17025. Data were obtained for 20 variables: temperature (°C), conductivity, pH, oxidability, total organic carbon, chloride, sulfur, sulfate, nitrogen, nitrate, ammonia, aluminum, barium, cobalt, copper, manganese, nickel, lead, selenium, and zinc.

**Figure 1:**
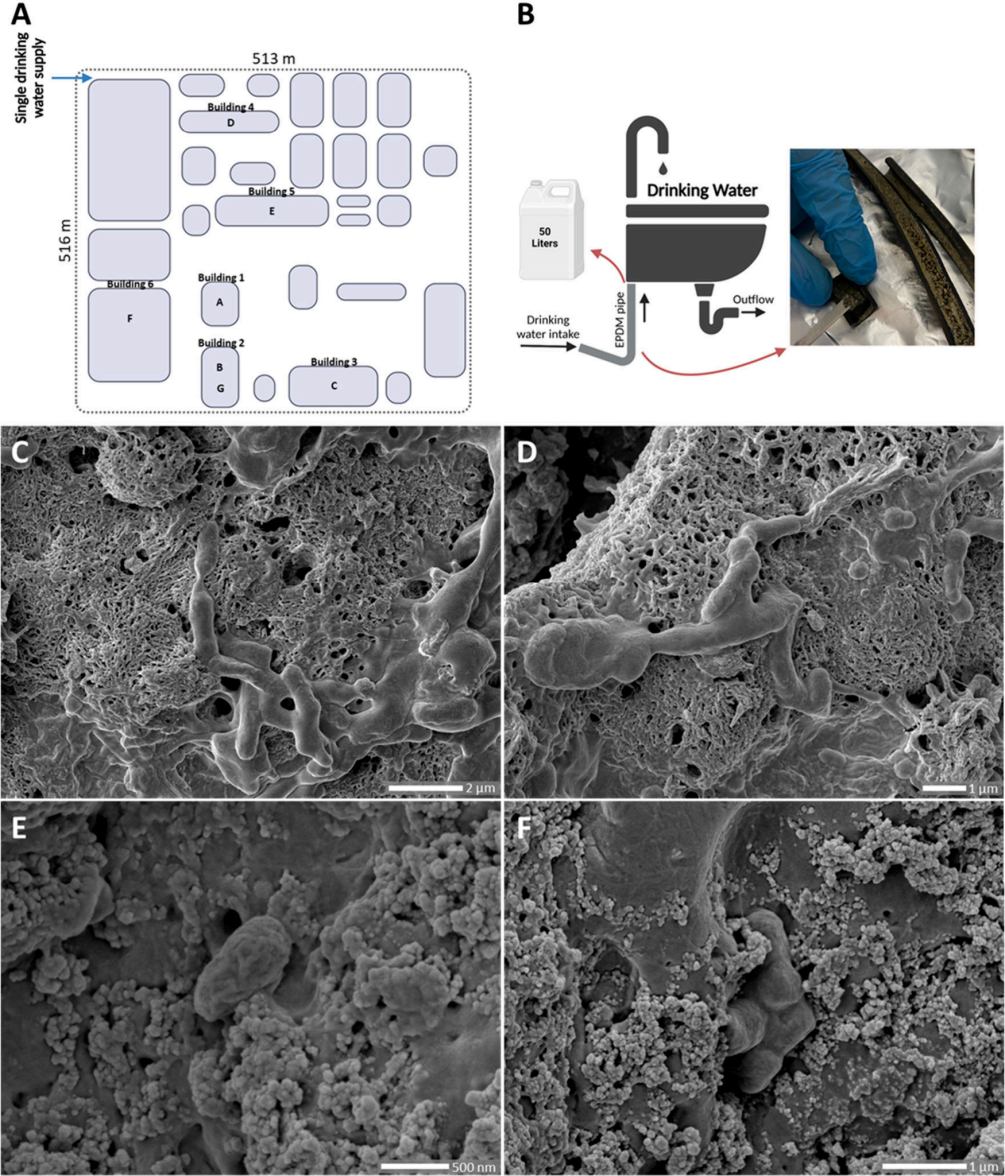
(A) Map of the buildings sampled in the DWDS, for which only one water entry exists. EPDM pipes B and G were located in the same washbasin, with segment G sampled one year after its installation (device G replaced device B). (B) Sampling strategy: after removing the EPDM devices, 50 L of drinking water was sampled and a new EPDM hose was installed. (C–F) Scanning electron microscopy images of biofilm F. Rod-shaped bacteria embedded in the biofilm matrix can be visualized in (C) and (D). Most parts of the EPS matrix observed in (C) and (D) presented a filamentous structure, while the matrix in (E) and (F) was more granular. In each microscopy image, the porosity of the matrix, which allows water to carry nutrients and other compounds to lower the cells through the formed channels, is observed.

### Cell and Virus-like Particle (VLPs) Counts in Water Samples

Ten mL water samples B, D and G were fixed with 4% formaldehyde (final concentration) and the fixation stopped by adding sterile phosphate-buffered saline (PBS) 1X. Four mL fixed samples were filtered through 0.22 μm pore-size isopore GTTP filters (Millipore), for cell counts, and 0.02 μm pore-size Anodisc (Whatman) filters, for VLP determinations. Subsequently, isopore GTTP filters were stained with 4′,6-diamidino-2-phenylindole (DAPI) and Anodisc filters were stained with SYBR^TM^ Gold (25X). Cells and VLP were counted on a Leica DM4000B epifluorescence microscope. In parallel, cell and VLP concentrations were also determined by flow cytometry. We followed the protocol described by Brussaard.^11^ Two mL of each selected water sample was fixed with glutaraldehyde (0.5% final concentration), frozen in liquid nitrogen, diluted in Tris-EDTA buffer (pH 8), stained with SYBR^TM^ Gold (0.5X final concentration), incubated at −80 °C, and maintained for 5 min at room temperature prior to analysis. The cytometer settings were as follows: the threshold was set in blue fluorescence (300 units), FITC voltage = 500, SSC voltage = 300, and forward scatter voltage = 500, and the flow rate was established as low. Background noise was checked on blanks, composed by TE buffer stained with SYBR^TM^ Gold. Samples were recorded at an event rate of 100–1000 events per second. Cells and VLPs counts were obtained by correcting the noise measured in blanks and expressed as cells/ml or VLPs/ml.

### Catalyzed Reported Deposition-Fluorescence in Situ Hybridization (CARD-FISH) and Scanning Electron Microscopy (SEM)

We followed the protocol described in Pernthaler et al.^12^ and adapted by Escudero et al.^13^ EPDM devices were cut in small pieces, without damaging the structure of the biofilm, and the samples were washed in ethanol 90% and air-dried. Cell permeabilization was determined with lysozyme and different hybridization and washing buffers were used depending on the probe (Table S2). Biofilms were counterstained with SYBR^TM^ Gold (25x), and preparations were mounted onto glass slides and covered with Citifluor (Citifluor, United Kingdom). The hybridized biofilms were observed with a ZEISS confocal laser scanning microscope model LSM 800, coupled with an Axio Imager Z.2 straight microscope and equipped with three laser diode excitation sources: 405 nm (UV), 488 nm (blue), 561 nm (green) and 640 nm (red). Images were collected with 63X oil immersion lens and the software ZEISS ZEN 3.9 was used to project the stacks to 2D images. As for SEM microscopy, first, the sample F was dehydrated with ethanol at different concentrations: 25% (5 min), 50% (5 min), 75% (5 min), 95% (5 min) and 100% (10 min). Then, a thin layer of platinum was sputtered onto the samples to enhance conductivity and prevent charging under the electron beam. The equipment used was a FESEM (Field Emission Scanning Electron Microscope) ZEISS model Merlin VP Compact, and the resolution was achieved at 5 kV.

### Characterization of the Biofilms’ Extracellular Polymeric Substances (EPS)

Biofilms with the highest biomass weight were used (D and F), and two strategies were followed for the detection and identification of sugars and other compounds: (i) total acid hydrolysis with trifluoroacetic acid (TFA) followed by High-Performance Anion-Exchange Chromatography with Pulsed Amperometric Detection (HPAEC-PAD); (ii) total acid hydrolysis with TFA followed by Gas Chromatography Mass Spectrometry (GC-MS).

Biofilms with the highest biomass were used (D and F). First, 300 mg of biofilm biomass was mixed with 1 mL of ultrapure water and sonicated for 15 min. After centrifuging at 10,000*g* for 10 min, the supernatant was freeze-dried. Part of the dry biomass (100 mg) was used for Nuclear Magnetic Resonance (NMR) analysis, and the rest of the sample for total acid hydrolysis with trifluoroacetic acid (TFA), following the indications described in Pettolino et al.^14^ For TFA hydrolysis, 100 μL of 2.5 M TFA was added to the dry samples and the mixture was incubated at 100 °C in a thermomixer, at 300 rpm for 4 h. Then, the preparations were cooled and placed at 30 °C, and the TFA was evaporated with a stream of nitrogen. Half of the hydrolyzed samples was used for High-Performance Anion-Exchange Chromatography with Pulsed Amperometric Detection (HPAEC-PAD) analysis, using different standards at 0.05 g/L (such as rhamnose, glucuronic acid, fucose, glucose, galactose, mannose, glycerol, maltose, glucosamine, fructose, sucrose, and *N*-acetyl-d-glucosamine). The compound separation was achieved at 30 °C for 25 min on a Dionex CarboPac PA-1 column (4 × 200 mm), equipped with its corresponding guard column, equilibrated with 4.5 mM NaOH, and running at a flow rate of 1 mL/min. The other half of the hydrolyzed samples was reduced and acetylated as explained in Pettolino et al.^14^ The final products were dissolved in 500 μL of dichloromethane (DCM) and transferred to a glass vial for further analysis by gas chromatography–mass spectrometry (GC-MS) in a GC-MS-Orbitrap Q Exactive system (Thermo Scientific).

### DNA Extractions, 16S rRNA Gene Amplification and Sequencing

DNA was extracted from biofilms and the 0.22-μm pore-size membranes using the FastDNA Spin Kit for soil (MP Biomedicals, Santa Ana, CA, USA), following the manufacturer’s recommendations, and fluorometrically quantified using Qubit 2.0 (Thermo Fisher Scientific) and High Sensitivity Assay (HS) kits. For 16S rRNA gene metabarcoding, the V3-V4 region of the 16S rRNA genes was PCR amplified with primers Pro341F/Pro805R.^15^ The PCR program employed was as follows: denaturation at 94 °C for 3 min, 30 cycles of denaturation (94 °C, 45 s), hybridization (51 °C, 1 min) and elongation (72 °C, 2 min), and a final extension at 72 °C for 10 min. PCR products were electrophoresed in a 1% agarose gel in TAE 1X buffer (40 mM Tris at pH 8.0, 20 mM acetic acid, 1 mM EDTA). PCR products were sequenced at FISABIO (Valencia, Spain) using Illumina Mi-Seq (2 × 300 bp). For metagenomes, extracted DNAs were sequenced at Macrogen (Seoul, South Korea) using a NextSeq500 (2 × 150 bp, 4 Gb/sample).

### Processing of 16S rRNA Gene Sequences

The 16S rRNA gene sequences from biofilm and water samples were analyzed with QIIME 2 2021.4.^16^ First, raw reads were demultiplexed and filtered by quality using the q2-cutadapt^17^ and q2-quality-filter^18^ plugins. Then, q2-vsearch,^19^ with the *de novo* clustering option, was used to generate Operational Taxonomic Units (OTUs) at 99% identity. Chimeras were removed with the option uchime-denovo, and final relative abundances were obtained with the plugin q2-feature-table; the option rarefy in this plugin was used for normalization according to the number of reads in the smallest library (sample WA_C, with 38,650 reads). Rarefaction curves were calculated and plotted with the Vegan Package^20^ in R v4.0.3 (www.r-project.org). Then, alpha diversity parameters (richness and Shannon’s indexes) were calculated using q2-diversity. Finally, the OTUs taxonomic affiliation was obtained by comparing the reference sequence of each OTU against the SILVA v138 SSU 16S rRNA database with the q2-feature-classifier^21^ and classify-sklearn^22^ with a confidence threshold of 70%.

### Analysis of Metagenomes

Metagenomic raw reads from water and biofilm samples were first processed with Trimmomatic v0.36 to remove the Illumina adapters and identify pair-end reads (LEADING:3 TRAILING:3 SLIDINGWINDOW:4:15 MINLEN:36).^23^ Then, the FastQC program was used to analyze the quality of the trimmed reads, the amount of Ns, the frequency of duplicated sequences and the presence of adapters.^24^ Second, the sequencing effort and the diversity of the metagenomes were determined with the Enveomics Nonpareil program.^25^ In parallel, reads belonging to 16S rRNA genes were extracted using SortMeRNA v4.3.6 ^26^ and taxonomically sorted by BLASTn against the SILVA v138.1 database,^27^ selecting only the best hit with the BlastTab.best_hit_sorted.pl option from Enveomics.^28^ Only those hits with coverages and identities higher than 70% and 83%, respectively, were considered.^29^

To assemble the metagenomic trimmed reads, several softwares were tested: IDBA-UD, metaSPAdes and MEGAHIT.^30–32^ Contigs obtained with metaSPAdes v3.13.0 presented the best quality; therefore, they were used for subsequent analyses. The prediction of Open Reading Frames (ORFs) and the extraction of the amino acid sequences were performed with Prodigal and the -p meta option.^33^

For the analysis of metabolic pathways, contigs were annotated with Anvi’o^34^ and the KEGG database.^35,36^ The completeness of each KEGG module and the number of KO-annotated proteins was obtained with “anvi-estimate-metabolism” and the options “--kegg-output-modes modules” and “--kegg-output-modes kofam_hits”. Only those KEGG modules with a completeness of ≥75% were considered. To verify that the observed differences between biofilms and water metagenomes were not attributed to the higher sequencing depth of the biofilm samples, the same analyses were performed after normalizing the metagenomes to the same number of sequences. In parallel, the standalone tool run_dbcan of dbCAN3 ^37^ was used to predict Carbohydrate-Active enZymes encoding genes (CAZymes).^38^ Run_dbcan is based on HMMER (e-value < 1e-15, coverage > 0.35) and DIAMOND BLASTp (e-value < 1e-102) analyses. Only those proteins correctly identified by both strategies were considered as CAZymes, as previously recommended.^39^

In addition, we specifically looked for those ORFs directly related to EPS synthesis and degradation. On the one hand, the detection of metabolic pathways and KOs involved in the production of exopolysaccharides (KEGG pathway map00543) and mycolic acids^40^ was performed. On the other hand, specific enzymes related to the synthesis or degradation of the most common exopolysaccharides detected in biofilms (such as alginate, Pel, Psl, cellulose, Poly-*N*-acetyl-glucosamine and xanthan, among others^41^), were searched by BLASTp (BlastTab.best_hit_sorted.pl, coverage > 50%, identity > 40%) against a homemade database containing 3,659 entries of glycosidases (EC 3.2.1.-: glycosidases, i.e. enzymes hydrolyzing O- and S-glycosyl compounds) and 535 entries of enzymes acting on polysaccharides (EC 4.2.2.-: lyases acting on polysaccharides), retrieved from the ENZYME repository^42^ and UniProtKB^43^ (Text S7).

Finally, proteins related to antibiotic resistance genes (ARGs) were annotated with the Resistance Gene Identifier (RGI) tool, based on the reference data from the Comprehensive Antibiotic Resistance Database (CARD),^44,45^ while Queuosine (Q) precursors genes were evaluated following the indications of Díaz-Rullo and González-Pastor,^46^ using the COG database, where these genes are referred as *queD* (COG0720), *queE* (COG0602), *queC* (COG0603), *queF* (COG0780 and COG2904), *tgt* (COG0343), *queA* (COG0809) and *queG/queH* (COG1600 and COG1636).

### Reconstruction and Analysis of Metagenome-Assembled Genomes (MAGs)

To generate metagenome-assembled genomes (MAGs), optimization of the binning process was first carried out. For the optimization, two different programs (MaxBin2 v2.2.7,^47^ and MetaBAT2 v2.15 ^48^) and several minimum contig sizes (1 kb, for MetaBAT2 only; 1.5 kb, for MaxBin2 only; 2 kb, 2.5 kb, 3 kb, 5 kb and 10 kb) were used. Completeness and contamination were determined for each MAG by CheckM2 ^49^ and for the subsequent analyses, the optimal contig size for each program and metagenome was selected based on the highest number of MAGs with completeness above 80% and contamination below 10%, considering that if the same number of MAGs with two different contig sizes were obtained, the largest contigs were selected. The selected MAGs were processed with the DAS Tool,^50^ that refines and selects nonredundant MAGs of good quality from a set of MAGs previously reconstructed with other binning algorithms. Then, in order to eliminate low quality MAGs, only those whose Q50 value, defined by [%completeness] – (5 × [%contamination]), was >50 were selected for further analysis.^51^ In addition, a manual refinement of the best quality MAGs (completeness > 80%) based on protein taxonomy of each contig (BLASTp vs NR database^52^) and their sequencing depth (coverage > 70%, identity > 95%) was performed, as previously described.^53^ Finally, MAGs were clustered in genomospecies at 95% ANI (Average Nucleotide Identity)^54^ with minimum aligned fraction of 30% (default value) using the dRep program,^55^ and functional annotations were performed as described above for metagenomic contigs, with the representative MAG.

The relative abundance of each genomospecies was estimated using the highest quality MAG within each genomospecies as the representative. A MAG was considered as detected in a given sample based on the value of breadth coverage calculated using samtools,^56^ assuming a positive detection if the value was greater than 10%.^57,58^ Then, for each metagenomic data set, the sequencing depth was estimated per position (Bowtie; BEDTools)^59,60^ and truncated to the central 80% values (i.e., removing the top and bottom 10% positions by depth; BedGraph.tad.rb),^28^ a metric hereafter termed TAD80 (truncated average sequencing depth). Relative abundances were then calculated as TAD80 normalized by the genome equivalents of the metagenomic data set (MicrobeCensus),^61^ resulting in units of community fraction. For the taxonomic classification of the genomospecies, the GTDB-tk v2.4.0,^62^ which is based on the alignments of 120 single-copy tagged genes, was used with the representative MAGs. Phylogenomic trees were constructed using PhyloPhlAn 3.0 ^63^ (function supertree_aa.cfg), and the corresponding amino acid identity (AAI) matrixes obtained (function supermatrix_aa.cfg). The final trees were visualized and drawn with iTOL.^64^

Finally, CRISPRCasFinder was implemented to detect CRISPR-Cas systems in the genomospecies, keeping only hits with levels 3 and 4 of confidence.^65^

### Detection and Analyses of Viral Contigs

Putative viral contigs ≥3 kb were extracted from the metagenomes with Vibrant^66^ and VirSorter2.^67^ Then, putative viral contigs were analyzed using the CheckV tool,^68^ which give a quality classification in addition to an estimation of the number of genes associated to phages or cells. Based on the obtained results, only contigs with more viral genes than host genes, and within the quality classification of “complete”, “high-quality”, “medium-quality” and “low-quality” were considered as “putative viral” contigs. Also, the viral genes of each putative viral contig had to represent at least 8% of their total predicted genes.^69^

Putative viral contigs which fulfilled these criteria were considered as viral contigs *bona fide* and were clustered into viral OTUs (vOTUs) based on their average nucleotide identity (ANI, ≥95%) and the alignment fraction (AF, ≥85%).^70^ The vOTUs were functionally annotated as described for contigs or MAGs, and taxonomically classified using the geNomad tool.^71^ The presence and relative abundances of the vOTUs in the different DWDS samples were assessed through read mapping of the trimmed reads from each metagenome by BLASTn, with a cutoff of 70% query coverage, e-value < 10^–1^ and the “best hit” option. Only those vOTUs mapped with identities ≥95% along ≥75% of the vOTUs reference sequence were considered.^72^ The *in silico* putative host assignation was conducted by the iPhoP tool,^73^ as well as with the protospacers of those CRISPR-Cas systems detected in the MAGs of this study against the vOTUs by BLASTn.^69^

Viral OTUs assigned to *Mycobacterium* were compared by BLASTn against all of the *Mycobacterium* isolated phages present in the NCBI Viral Genomes Resource. In addition, vConTACT2 ^74^ was implemented to assess the relationships between the *Mycobacterium*-associated vOTUs and all the *Mycobacterium* isolated phages. The tool utilizes a gene content-based network approach to cluster viral genomes into related groups based on shared protein families. The resulting viral genome similarity network was visualized using Cytoscape (version 3.9.1).^75^

### Statistical Analyses

Statistical differences in physicochemical characteristics between the different water samples were calculated with PERMANOVA^76^ (permutations: 4999, method: “bray”) after checking the homogeneity of variance with “betadisper” from the Vegan Package^20^ in R v.4.0.3, while statistical differences in Alpha diversity parameters (Richness and Shannon’s index) in the whole data set were calculated using the Stats Package in R and plotted using ggplot2.^77^ Normality was examined using the Shapiro-Wilk test, and nonparametric tests (Mann–Whitney U Test and Kruskal–Wallis Test) were applied to the Alpha diversity values of the different sample types (biofilm or water).

Statistically significant differences of the OTUs distribution in waters and biofilms were also evaluated using PERMANOVA^76^ (permutations: 4999, method: “bray”) and represented using a using nonmetric multidimensional scaling (NMDS) based on a Bray–Curtis distance calculation with the Vegan Package^20^ in R. SIMPER analysis, included in the Vegan Package,^20^ was used to determine “prevalent” bacteria within the data sets, those with a relative abundance statistically higher (p-value < 0.05) under a certain condition. As for metabolic traits differences, the SIMPER method was also employed to identify any of the detected functions from the various databases used for the metagenomes and MAGs annotation that showed significant differences among the conditions (sample type, biofilm stage).

## Results and Discussion

### Sampling Characteristics and Physicochemical Properties

Seven EPDM rubber hoses (A–G) that connected the main DWDS from six buildings to individual washbasins were sampled (Figure 1A,B). While the time of use of segments A–F could not be determined (not even by considering certain biofilm characteristics such as the amount of recovered biomass, because of inorganic deposits mixed with microbes), segment G was sampled one year after its installation. The chlorinated water circulating through these devices showed no statistically significant physicochemical differences (Table S3), including the water sample taken one year later. Cell and VLP concentrations were in the range of 10^4^/mL (Table S4), consistent with previous studies.^4^

Biofilms with the highest microbial biomass (D and F) were visualized under SEM (Figure 1C–F) and were also used to chemically characterize their EPS matrix. No sugars could be identified by Mass Spectrometry, and although various peaks were observed by HPAEC-PAD, only glycerol could be clearly detected (Figure S1). Albeit glycerol itself is not a backbone component of exopolysaccharides, it can be involved in the biofilm matrix as a metabolic byproduct or as part of glycerol-containing lipids, such as the *Mycobacterium* mycolic acids.^78^

### Metataxonomic Study of the Selected DWDS: Biofilms and Planktonic Fractions

Amplicons of 16S rRNA genes were obtained for all water and biofilm samples, yielding 1,384,422 high-quality reads which were clustered into 1,321 OTUs at 99% identity (to refer to the amplicon sequences of each sample, prefixes “BA”, biofilm amplicon, or “WA”, water amplicon, were added before the sample names). Rarefaction curves reached a plateau in all cases, indicating that the sequencing effort captured most of their microbial diversity (Figure S2), and Good’s coverage, which calculates the proportion of nonsingletons,^79^ was above 99.8%.

Alpha diversity was computed using the number of different OTUs (richness) and the Shannon diversity index (Figure 2A,B), with no statistically significant differences between biofilms and water samples. Shannon’s indexes were also used to determine the degree of biofilm maturity (Figure 2B), as in previous works,^80,81^ since no other characteristics of the samples could be used. As expected, the lowest Shannon value corresponded to biofilm G, whose biomass was collected one year after the EPDM hose placement. Consequently, we catalogued biofilm G as “incipient”, biofilms A, C and D as “intermediate” and B, E and F as “old” biofilms. Regarding beta diversity, an NMDS ordination based on OTU relative abundances of the OTU showed that most samples clustered according to their origin (biofilm or water) (Figure 2C). This separation was further supported by PERMANOVA analysis (p = 0.019). Indeed, only 24.4% of the OTUs were detected in both sample types. At the genus level, a higher proportion of OTUs (43%) belonged to genera shared between water and biofilm samples (Tables S5 and S6). However, these shared genera accounted, on average, for up to 65% of the reads in biofilm samples but only 26% in water samples. Given that no significant differences in the physicochemical parameters of the water were observed among the studied DWDS samples, even one year later, the observed differences in beta diversity between waters and biofilms might be explained by the presence of planktonic bacteria that do not participate in biofilm formation as well as bacteria detached from biofilms developed upstream on different materials.

**Figure 2:**
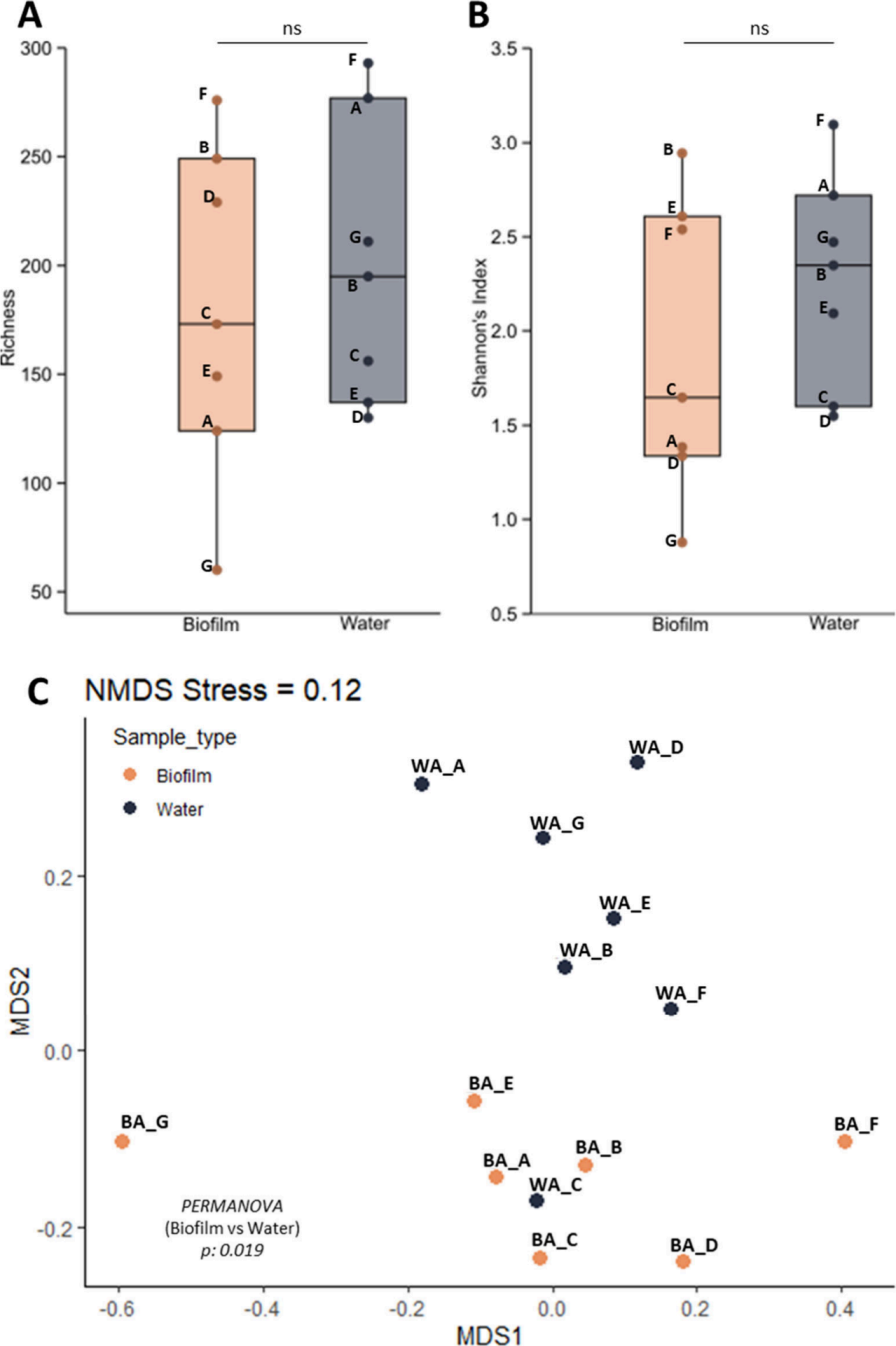
(A,B) Alpha diversity metrics (*y*-axis) derived from the 16S rRNA gene metabarcoding analysis from biofilm and water samples (*x*-axis). (C) Nonmetric multidimensional scaling (NMDS) analysis based on the relative abundances of the OTUs from biofilm and water samples. Before each sample name, the preffix “BA_” (biofilm amplicon) or “WA_” (water amplicon) was added.

Overall, water and biofilm samples were dominated by members of *Bacteria* (Table S5), with *Pseudomonadota*, *Actinomycetota*, *Cyanobacteriota*, *Bacteroidota* and *Planctomycetota* as the phyla with the highest averaged relative abundances (Figure 3A). Statistical analyses were performed to distinguish between “generalist” bacterial genera in the DWDS (present in ≥ 50% of both biofilm and water samples with no significant differences in their relative abundances between both types of samples), “specific” genera (present in ≥ 50% of biofilm or water samples), or “prevalent” genera (those with statistically higher relative abundances in biofilms or water) (Figure 3B; Table S6). Only *Mycobacterium*, *Chryseolinea* and *Amphiplicatus*, all of them previously detected in other water facilities,^7,82,83^ fell into the category of “generalist”. They were also “abundant”, since their relative abundances were ≥1% (in an analogous way to the definition of abundant biosphere by Pedrós-Alió^84^) in ≥50% of the samples (Figure 3B). *Mycobacterium*, able to survive and develop in treated waters and considered as an emerging pathogen of concern for water utilities,^85^ was not detected in the incipient biofilm G, but it was present in all water samples and in the rest of biofilms with high relative abundances. To our knowledge, there are no studies describing *Mycobacterium* as a surface colonizer; however, mycobacteria are known to promote biofilm formation and to dominate established biofilms.^10,86^

**Figure 3:**
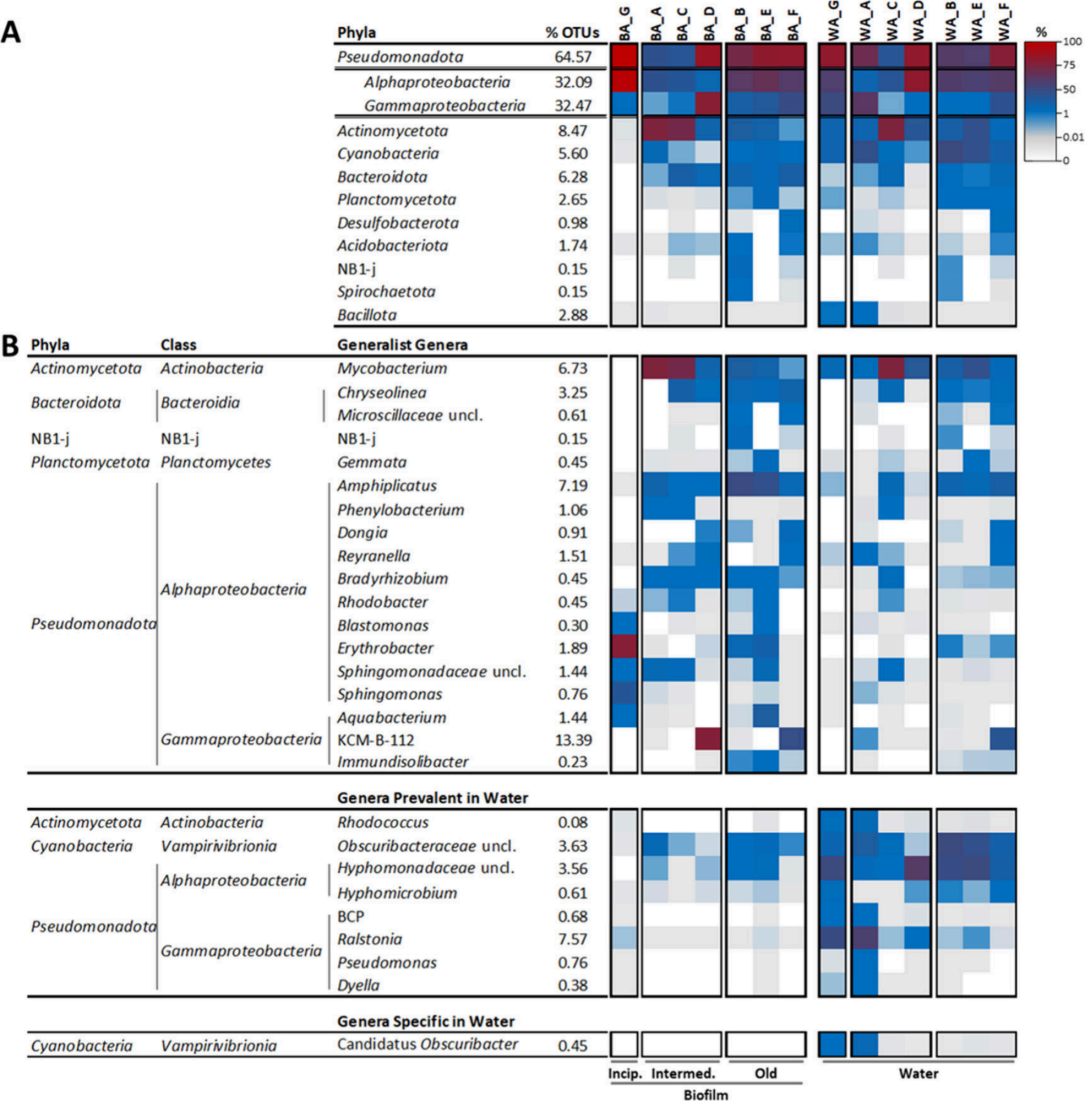
Heatmap of the bacterial phyla (A) and genera (B) that exceeded 1% relative abundance in at least one biofilm or water sample. Those genera present in ≥50% of both biofilms and water samples, with no significant differences in their relative abundances between both types of samples, were considered as “generalist”, while those genera present in biofilms and water samples, with a relative abundance statistically higher in waters were considered as “prevalent in water”. Genera exclusively present in ≥50% water samples were considered as “specific in water”. Genera “prevalent in biofilms” or “specific in biofilms” are not shown as none of them exceeded 1% of relative abundance in any sample. Before each sample name, the prefix “BA_” (biofilm amplicon) or “WA_” (water amplicon) was added.

In water samples, 5 and 9 genera were specific or prevalent, respectively, and commonly found in aquatic environments, including DWDS.^87,88^ Among the specific genera, only the cyanobacterium *Candidatus obscuribacter* exceeded 1% relative abundance in at least one sample (Figure 3B). In biofilms, 3 genera were specific and 2 were prevalent; in terms of relative abundances, all of them showed values < 1%. However, in the incipient biofilm G high relative abundances of some generalist genera were found, such as *Erythrobacter*, *Sphingomonas*, *Aquabacterium* and *Blastomonas*, which include biofilm-forming species (Figure 3B).^89–92^ As the biofilm’s maturity increased, 5 generalist genera (*Amphiplicatus*, *Dongia*, *Gemmata*, *Ketobacter* and *Piscinibacter*) increased their relative abundances, exceeding 1% in the oldest biofilms. Remarkably, the genus *Pseudomonas*, ubiquitous in DWDS,^3,10,93^ was only detected in two biofilms with <0.1% of relative abundance. Its low relative abundance could be due to the pipe material since *Pseudomonas* biofilms are preferably developed on ductile iron pipes rather than polyvinyl chloride, polyethylene or steel materials.^3,94^

### In Situ Distribution of Phyla in DWDS Biofilms

CARD-FISH was used on the biofilms with the highest amount of biomass (B-F) to obtain information about the spatial organization of the main bacterial phyla, in terms of relative abundance, within the biofilm matrix (Figure 4). Our results showed no specific phyla aggregation or distribution, but a general presence all over the biofilms suface. Detected bacteria were estimated from around 3.6 × 10^3^ cells/mm^2^ (for *Actinomycetota* and *Bacteroidota*) to 1.0 × 10^3^ cells/mm^2^ (for *Planctomycetota* and *Cyanobacteria*). Members of the classes *Alphaproteobacteria* and *Gammaproteobacteria* were observed with a frequency around 2.0 × 10^3^ and 1.2 × 10^3^ cells/mm^2^, respectively.

**Figure 4:**
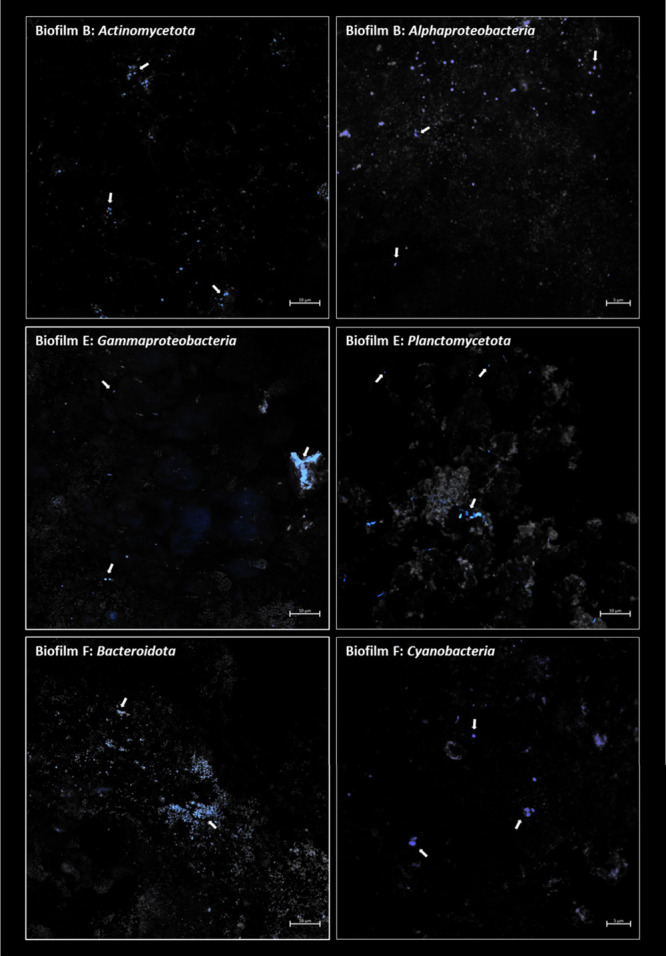
CARD-FISH images of those phyla with the highest relative abundances in biofilms. Each image is the result of the composite of those cells hybridized with a given probe (in blue, white arrows) and the rest of cells stained with SYBR^TM^ Gold (in gray).

### Functionality in the Selected DWDS through Metagenomics

The microbial functional patterns from water and biofilm communities were analyzed in all the biofilms and 3 water samples by metagenomics (DNA amounts extracted from water samples A, D, E and G were not enough for sequencing). All metagenomes were of high quality, based on Nonpareil data (Table S7) and the taxonomic composition of the metagenomic reads, as well as the diversity parameters, were consistent with those values obtained in the metataxonomic analysis (Table S8).

Open reading frames (ORFs) were predicted from contigs ≥1 kb and protein sequences were annotated with the KEGG database^95^ (Table S9). The percentage of genes associated with KEGG Orthologs in each metagenome ranged from 40.16% to 48.69%. No statistically significant differences were observed between biofilm and water samples at the different KEGG levels; however, the number of metabolic pathways reconstructed was higher in biofilms (211) than in water (187). For the three biofilm stages that number was in concordance with their degree of maturity (86 pathways in the incipient biofilm, 194 in intermediate biofilms, and 205 in old biofilms) (Table S9).

To obtain information about biofilm dynamics, we searched for genes related to biofilm evolution and virulence, such as those involved in queuosine synthesis, EPS metabolism, and ARGs (Table S9). Queuosine is a modified nucleoside which is incorporated into tRNAs to enhance the expression of Q-genes, those enriched in NAU codons and that control various processes in bacteria, such as cell adhesion, biofilm formation or virulence.^46^ In the studied DWDS, genes for the synthesis of queuosine were detected at significantly higher abundances in biofilms than in the water samples and also in the intermediate/old biofilms compared to the incipient one (Table S9). With respect to the EPS, the biosynthesis of complex exopolysaccharides involves the action of different glycosyltransferases (GTs).^96^ Genes encoding GTs from the families GT2 and GT4, predicted to act in the synthesis of specific polysaccharides such as those contained in colanic acid, alginate, Psl, Pel, PNAG, xanthan, succinoglycan, and cellulose, were found in the metagenomes of all biofilms, with relative abundances which were proportional to the biofilm maturity (Figure S3; Table S9). However, since these CAZymes families are not specific enough, we searched for the genes involved in the synthesis of the abovementioned exopolysaccharides. In the incipient biofilm, no complete pathways could be reconstructed (Figure S3), consistent with the fact that these functions become more complex as biofilm gains in microbial diversity.^97,98^ KOs for the synthesis of *Mycobacterium* mycolic acids, important in the formation of hydrophobic extracellular matrices,^99^ were detected in all intermediate and old biofilms, where *Mycobacterium* was abundant (Table S9).

Antibiotic resistance genes (ARGs) were also detected in all metagenomes, except in the incipient biofilm, with their highest proportions in old biofilms (Figure S3; Table S9). Our observation was consistent with the literature^100^ and highlights the importance of frequent DWDS treatments, such as Ice Pigging, to mitigate the biofilm development which can converge toward an increase in the proliferation and dispersion of antibiotic resistance mechanisms. Indeed, although the Drinking Water Directive does not set specific parameters for antibiotic resistance bacteria (ARB) or ARGs as contaminants with limit values, it does introduce new requirements that are relevant to the fight against antimicrobial resistance. Specifically, the Directive “adopts a One Health approach where all the emerging contaminants should be analyzed, including ARB or ARGs”.

Among the detected ARGs, the most abundant was the gene *adeF*, which is a membrane protein of the AdeFGH multidrug efflux complex. This complex is related with resistance against tetracyclines, among other antibiotics.^101^ However, we must be cautious since the complete operon was not detected and, in any case, the role of the AdeFGH complex in antibiotic resistance seems to depend on its level of expression, rather than in its mere presence.^102^ Other relevant detected ARGs were those coding for the OXA-50 protein (a beta-lactamase)^103^ and an erythromycin-resistance rRNA methylase.^104^ In the old biofilms, complete pathways for the synthesis of kanosamine (an antibiotic which can also be part of more complex antibiotics such as kanamycin^105^) were detected, as well as genes for the synthesis of pyrrolnitrin (a secondary metabolite with antifungal and antibacterial properties^106^) (Table S9).

### Metagenome-Assembled Genomes: “Who” Does “What”

Community metagenomics does not easily assign functions to specific taxa, so, to unveil the role of certain microbes within the DWDS, a total of 185 high- and medium-quality MAGs (≥80% completeness, < 5% contamination) were reconstructed and clustered in 104 genomospecies (ANI ≥ 95%) (Table S10). All genomospecies accounted for 30.9–61% of the reads in each metagenome (Figure 5A) and represented 22.4% of the OTUs obtained in the metataxonomic study using an identity threshold above 98.6%^29^ (Table S10), so they were considered as good representatives of the total genomic diversity of the samples. For biofilm G and the water sample B, with the lowest diversities, most reads were recruited by only one genomospecies. Indeed, only one MAG could be assembled from incipient biofilm G, whose reads did not map against any other genomospecies.

**Figure 5:**
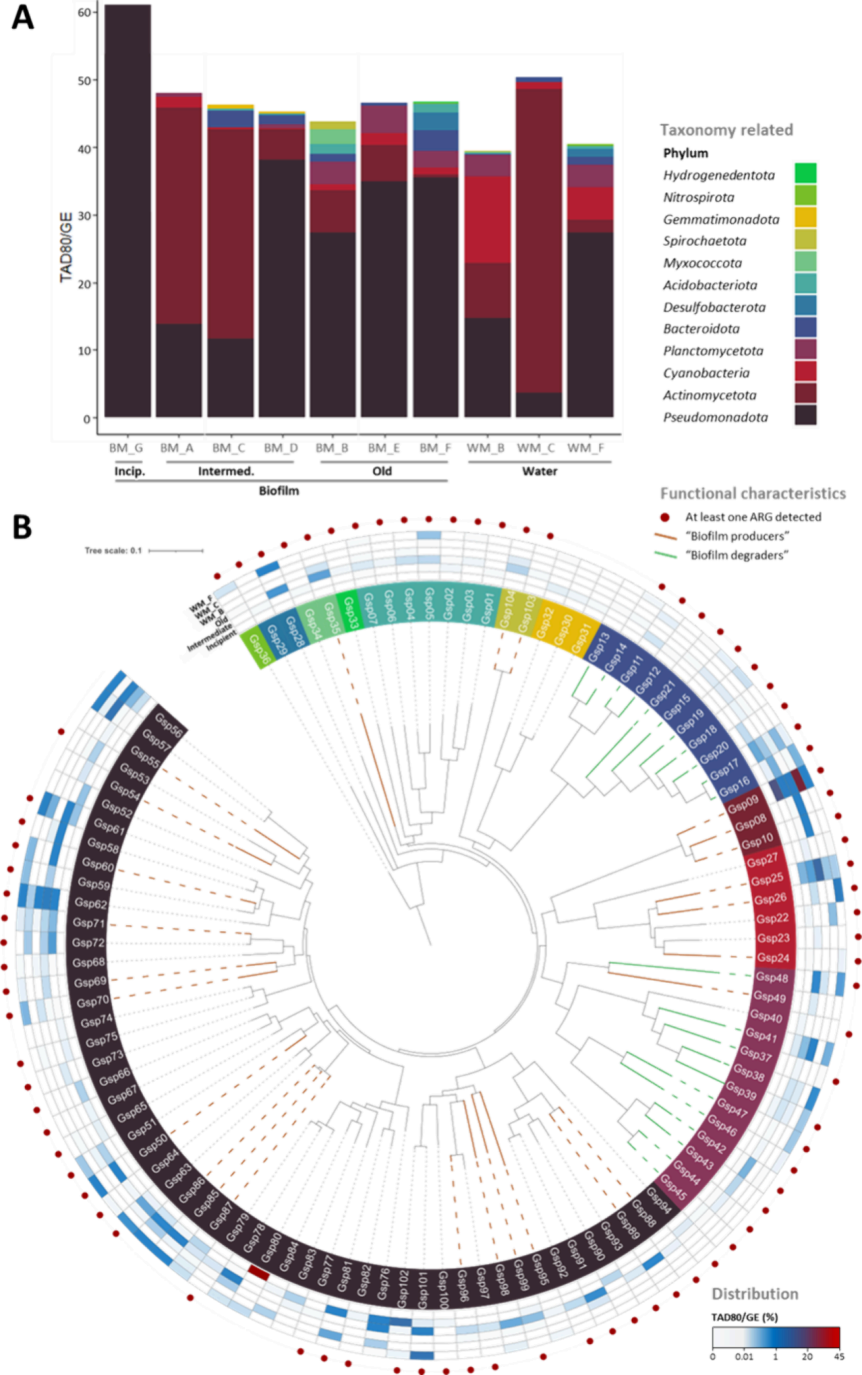
(A) Cumulative relative abundance (TAD80/GE; *y*-axis) of bacterial phyla in the DWDS metagenomes (*x*-axis) based on the genomospecies composition. (B) Phylogenomic tree of the obtained 104 genomospecies. In (B), the layers over the genomospecies names are a heatmap of the relative abundance of each genomospecies in the different biofilm stages (incipient, intermediate, and old) and the water samples. For the relative abundances in intermediate and old biofilms, average value were calculated with the data of the 3 metagenomes of each stage. Red dots indicate the presence of, at least, one ARGs in each genomospecies. Branch colors are associated with genomospecies considered as “biofilm producers” (in brown) or the main “biofilm degraders” (in green; those with ≥1% of their genes associated with EPS degradation).

MAGs were classified in 12 bacterial phyla (Figure 5B). Remarkably, 95% of the genomospecies belonged to unclassified taxa below the family level, indicating that the DWDS is a source of taxonomic novelty. Six genomospecies, affiliated to *Mycobacterium gadium* (Gsp09), genus *Bradyrhizobium* (Gsp71) and families *Parvularculaceae* (Gsp62), *Sphingomonadaceae* (Gsp76), *Obscuribacteraceae* (Gsp25) and *Hyphomonadaceae* (Gsp56), were ubiquitous and abundant in the system, as also determined in the metataxonomic study (Figure 5B).

Seventy-eight percent of the genomospecies harbored at least one ARG. The resistances against a broader range of antibiotics were present in members of *Cyanobacteriota*, *Actinomycetota*, *Acidobacteriota* and *Gammaproteobacteria*, while some taxonomic groups, such as *Gemmatimonadota*, the UBA11222 order within *Alphaproteobacteria* or the *Sphingorhabdus* and *Immundisolibacter* genera, among others (Figure 5B; Table S11), did not present any ARG (or no contigs with ARGs were binned for these MAGs). As previously mentioned, *adeF* was the most abundant ARG, while an RNA-polymerase binding protein (RbpA), which confers resistance to rifampin,^107^ and the *Mycobacterium tuberculosis* gene *murA*, that confers intrinsic resistance to fosfomycin,^108^ were found in the genomospecies affiliated to *Mycobacterium*. The wide distribution of ARGs among our genomospecies is consistent with the correlation between chlorine pressure and antibiotic resistance emergence.^109^

Genes for queuosine synthesis were detected in 65% of the genomospecies (Q-sources), mainly members from *Pseudomonadota*, and some genomospecies were associated with the biosynthesis of antibacterial compounds or with the degradation of some potential contaminants. The syntheses of beta-lactams (cephamycin C), fosfomycin, and pyrrolnitrin were the most represented pathways, although incomplete, within members from *Pseudomonadota*, *Acidobacteriota*, *Actinomycetota* and *Bacteroidota*. Remarkably, 34 genomospecies from 8 phyla, well distributed among the different biofilm stages, presented genes *merA* and/or *merB*, which participate in the methylmercury detoxification (Table S11).^110^ Also, complete metabolic pathways for the degradation of other contaminants that can impact the quality of the water were found, such as catechol, phenylacetate, and phthalate, among others. Phthalates are widely recognized as endocrine-disrupting compounds^111^ and phenylacetate is a chlorination byproduct which is associated with undesirable effects.^112^ Catechol is not commonly studied as a direct contaminant in DWDS, but it can contribute to biofilm formation and act as a precursor for various disinfection byproducts.^8^ Such degradation pathways were generally found in genomospecies, which were more abundant in old biofilms and classified within *Pseudomonadota*, *Actinomycetota* and *Gemmatimonadota*.

All genomospecies seemed to be involved in the evolution of the EPS matrix. Those genomospecies with more ORFs related to EPS formation rather than EPS degradation were considered as “biofilm producers” (22% of the genomospecies), while those showing the opposite trend (72%), were considered as “biofilm degraders”. Biofilm producers mainly affiliated with *Pseudomonadota* and *Cyanobacteriota* (Figure 5B; Figure S4) and harbored at least one gene related to the synthesis of alginate, Pel, Psl, succinoglycan or colonic acid and, as expected, KOs for mycolic acid production were detected in the three *Mycobacterium* genomospecies (Table S11). Classes *Bacteroidia* and *Planctomycetes*, well-known polysaccharides degraders,^113,114^ contained genomospecies with ≥1% of their genes associated with EPS degradation (Figure 5B), with Gsp20 (order *Cytophagales*) harboring the highest proportion of genes associated with polysaccharides degradation (2.3%) and half of them directly related to the degradation of alginate, Pel and Psl. In general, most “biofilm degraders” and “biofilm producers” increased their abundances along with biofilm maturity.

In summary, the formation and evolution of biofilms are associated with heterogeneous microbial communities, which pose a challenge to the routine maintenance of DWDS, as they contribute to water quality deterioration, increased disinfectant demand, and health risks. The presence of potential pathogens and ARGs may represent suitable targets for the development of molecular diagnostic tools to support rapid decision-making within Water Safety Plans (WSPs; Environment, Climate Change and Health [ECH], 2022). Additionally, strategies aimed at preventing biofilm formation and thereby reducing the release of microbial cells into the water flow are critical for maintaining water quality.

### Insights into the DWDS Viral Population Diversity and Dynamics

The diversity and impact of viruses in DWDS remains underexplored despite their role either killing biofilm microbial producers and potentially pathogenic and antimicrobial resistant bacteria, or disrupting the biofilm matrix to better expose microbes to the effect of viruses or disinfectants.^115^

In this study, more than 1,000 viral contigs extracted from water and biofilm metagenomes were clustered in 712 vOTUs, with more than 80% of the viral contigs from biofilms and an increase in their recovery as biofilms mature (Table S12). The vOTUs recruited up to 6.11% of biofilms reads and most of them (96%) were assigned to the class *Caudoviricetes* (Figure S5), which contain tailed viruses. None of them could be classified at the genus/species level, underscoring the taxonomic novelty of the DWDS viral assemblages.^116^ Considering all of the reads that mapped against the vOTUs (the set of detected viral reads within each metagenome), *Caudoviricetes* recruited 98 and 99.9% of them in biofilms and water metagenomes, respectively.

The IPhoP software assigned 171 vOTUs to 55 bacterial genera detected in the studied DWDS, including *Mycobacterium*, *Bradyrhizobium*, *Bosea*, *Sphingopyxis* or *Erythrobacter*, among others (Table S12). In addition, the search of 1,602 CRISPR-Cas systems spacers, extracted from 29 genomospecies, against the vOTUs, allowed for the association of 2 vOTUs to Gsp85 (*Alphaproteobacteria* - UBA11222) and Gsp89 (*Aquabacterium commune*) (Figure S6). In general, the relative abundances of those vOTUs and hosts in the abundant biosphere were positively correlated (p-value < 0.05). The correlation was very clear in the case of *Mycobacterium* and their associated viruses. *Mycobacterium* dominated the microbial community in intermediate biofilms (21.9% of 16S rRNA reads in average) and was linked to vOTUs that recruited >60% of the viral reads in those samples. Half of the viral reads were recruited by vOTUs with integrases (putative prophages). Remarkably, although *Mycobacterium* is among the bacterial genera with the best described virosphere,^117^ only 5 vOTUs presented a direct gene-sharing network with described mycobacteriophages, which reinforced the presence of an important viral novelty in this type of environment (Figure S7).

Since phages are also known for their roles as “EPS degraders”,^115^ some specific genes, such as viral depolymerases, were looked for in our set of vOTUs. Putative CAZyme encoding genes were predicted in 11% of the vOTUs. In most of them (57 vOTUs) they were related to peptidoglycan degradation (GH19, GH23, GH24, GH73, GH104, GH108), while 11 vOTUs harbored enzymes that could act against alginate compounds (Table 1). In addition, *lysB* genes (Lysin B) were detected in three vOTUs, with two of them (vOTU67 and 84) assigned to *Mycobacterium* viruses. Lysin B is a mycolylarabinogalactan esterase that presents antimycobacterial activities and is predominantly associated with mycobacteriophages. This enzyme facilitates the host lysis by compromising the integrity of the mycobacterial outer membrane.^118^ Also, Lysin B has shown promising activity against multidrug resistant strains of *M. tuberculosis*, suggesting a potential role as a therapeutic agent in combination with other treatments.^119^ Although phage therapy remains limited by regulatory barriers, research on phage-host interactions within DWDS biofilms offers new insights into its potential effectiveness for biofilm control. The application of phages or viral enzymes is emerging as a promising approach that could be integrated into the development of modern Water Safety Plans.

**Table 1:** CAZy Families Predicted among the vOTUs^a^

Predicted CAZy families	Predicted CAZy activities	vOTU id	Family	Putative host
CE5	Triacylglycerol lipase (EC 3.1.1.3); Acetylxylan esterase (EC 3.1.1.72); Cutinase (EC 3.1.1.74)	vOTU67*	*Caudoviricetes*	Genus *Mycobacterium* (*Actinomycetota*)
		vOTU84*	*Caudoviricetes*	Genus *Mycobacterium* (*Actinomycetota*)
				
GH104	Lysozyme (EC 4.2.2.29)	vOTU41	*Caudoviricetes*	Genus *Bosea* (*Pseudomonadota*)
				
GH108	Lysozyme (EC 3.2.1.17)	vOTU523	*Caudoviricetes*	Genus *Bosea* (*Pseudomonadota*)
		vOTU112	*Caudoviricetes - Crassvirales*	Genus ELB16-189 (*Bacteroidota*)
		vOTU729	*Caudoviricetes*	Genus *Halomonas* (*Pseudomonadota*)
		vOTU503	*Caudoviricetes*	Genus *Hyphomicrobium* (*Pseudomonadota*)
		vOTU391	*Caudoviricetes*	Genus *Sphingobium* (*Pseudomonadota*)
		vOTU529	*Caudoviricetes*	Genus *Sphingobium* (*Pseudomonadota*)
		vOTU172	*Caudoviricetes*	
		vOTU235	*Caudoviricetes*	
		vOTU329	*Caudoviricetes*	
		vOTU390	*Caudoviricetes*	
		vOTU467	*Caudoviricetes*	
				
GH113	Mannosidase (EC 3.2.1.-; 3.2.1.25; 3.2.1.78)	vOTU30	*Caudoviricetes*	
		vOTU641	*Caudoviricetes*	
				
GH16	Glucosidase (EC 3.2.1.6,21,39,58,73,151,...); Galactosidase (EC 3.2.1.23,81,83,103,181,...·)	vOTU96	*Caudoviricetes*	Genus *Mycobacterium* (*Actinomycetota*)
				
GH18	Chitinases (EC 3.2.1.-,14,200,201); Mannosyl-oligosaccharide endo-b-1,4-N-acetylglucosaminidase (EC 3.2.1.96)	vOTU559	*Caudoviricetes*	-
				
GH19	Lysozyme (EC 3.2.1.17); Chitinase (EC 3.2.1.14)	vOTU733	*Caudoviricetes*	Genus 2-12-FULL-53-21 (*Desulfobacterota*)
		vOTU531	*Caudoviricetes*	Genus *Bosea* (*Pseudomonadota*)
		vOTU417	*Caudoviricetes*	Genus *Bradyrhizobium* (*Pseudomonadota*)
		vOTU646	*Caudoviricetes*	Genus *Reyranella* (*Pseudomonadota*)
		vOTU680	*Caudoviricetes*	Genus *Reyranella* (*Pseudomonadota*)
		vOTU20	*Caudoviricetes*	
		vOTU405	*Caudoviricetes*	
		vOTU477	*Caudoviricetes*	
		vOTU657	*Caudoviricetes*	
		vOTU674	*Caudoviricetes*	
				
GH23	Lysozyme (EC 3.2.1.17, 4.2.2.29); Chitinase (EC 3.2.1.14)	vOTU153	*Viruses Uncl*.	Genus *Mycobacterium* (*Actinomycetota*)
		vOTU307	*Caudoviricetes*	Genus *Mycobacterium* (*Actinomycetota*)
		vOTU338	*Caudoviricetes*	Genus *Mycobacterium* (*Actinomycetota*)
		vOTU72	Viruses Uncl.	Genus *Mycobacterium* (*Actinomycetota*)
		vOTU81	*Caudoviricetes*	Genus *Mycobacterium* (*Actinomycetota*)
		vOTU33	*Caudoviricetes*	Genus *Sphingopyxis* (*Pseudomonadota*)
		vOTU68	*Caudoviricetes*	
		vOTU701	*Caudoviricetes*	
		vOTU99	*Caudoviricetes*	
				
GH24	Lysozyme (EC 3.2.1.17)	vOTU670	*Caudoviricetes*	Genera *Altererythrobacter* and *Erythrobacter* (*Pseudomonadota*)
		vOTU310	*Caudoviricetes*	Genus *Cereibacter* (*Pseudomonadota*)
		vOTU466	*Caudoviricetes*	Genus *Cereibacter* (*Pseudomonadota*)
		vOTU42	*Caudoviricetes*	Genus *Erythrobacter* (*Pseudomonadota*)
		vOTU737	*Caudoviricetes*	Genus *Erythrobacter* (*Pseudomonadota*)
		vOTU716	*Corticoviridae (Tectiliviricetes)*	Genus Ga0077527 (*Pseudomonadota*)
		vOTU734	*Caudoviricetes*	Genus *Reyranella* (*Pseudomonadota*)
		vOTU683	*Caudoviricetes*	Genus *Roseococcus* (*Pseudomonadota*)
		vOTU32	*Caudoviricetes*	Genus *Sphingopyxis* (*Pseudomonadota*)
		vOTU414	*Caudoviricetes*	Genus *Tabrizicola* (*Pseudomonadota*)
		vOTU78	*Caudoviricetes*	Genus *Tropicimonas* (*Pseudomonadota*)
		vOTU28	*Caudoviricetes*	
		vOTU29	*Caudoviricetes*	
		vOTU341	*Caudoviricetes*	
		vOTU394	*Caudoviricetes*	
		vOTU437	*Caudoviricetes*	
		vOTU446	*Caudoviricetes*	
		vOTU491	*Caudoviricetes*	
		vOTU512	*Caudoviricetes*	
		vOTU544	*Caudoviricetes*	
		vOTU630	*Caudoviricetes*	
		vOTU649	*Caudoviricetes*	
		vOTU725	*Caudoviricetes*	
		vOTU361	*Caudoviricetes*	
		vOTU379	*Caudoviricetes*	
				
GH39	Cellulose glucosidase (EC 3.2.1.74); Arabinofuranosidase (EC 3.2.1.215); Rhamnosidase (EC 3.2.1.-); Xylosidase (EC 3.2.1.37)	vOTU115	*Caudoviricetes*	Genus *Mycobacterium* (*Actinomycetota*)
		vOTU303	*Caudoviricetes*	Genus *Mycobacterium* (*Actinomycetota*)
		vOTU358	*Caudoviricetes*	Genus *Mycobacterium* (*Actinomycetota*)
				
GH51	Cellulose glucosidase (EC 3.2.1.4,91); Arabinofuranosidase (EC 3.2.1.55,215); Xylosidase (EC 3.2.1.8,37)	vOTU381	*Caudoviricetes*	
				
GH73	Lysozyme (EC 3.2.1.-); Mannosyl-oligosaccharide endo-b-1,4-N-acetylglucosaminidase (EC 3.2.1.96)	vOTU661	*Caudoviricetes*	
				
GT101	Hexosyltransferases (EC 2.4.1.-)	vOTU352	*Caudoviricetes*	
				
GT102	N-acetylglucosaminyl-PP-decaprenol α-1,3-L-rhamnosyltransferase (EC 2.4.1.289)	vOTU554	*Viruses Uncl*.	
				
GT2	Glycosyltransferase (2.4.1.-,12,16,117,157,212,···)	vOTU114	*Caudoviricetes*	Genus *Bradyrhizobium* (*Pseudomonadota*)
		vOTU546	*Viruses Uncl*.	
		vOTU619	*Caudoviricetes*	
				
GT25	Galactosyltransferase (EC 2.4.1.-); Xylosyltransferase (EC 2.4.2.-); Acetylgalactosaminyltransferase (EC 2.4.1.-)	vOTU40	*Caudoviricetes*	
				
GT32	α-glucosyltransferase (EC 2.4.1.-)	vOTU1	*Caudoviricetes*	
		vOTU550	*Caudoviricetes*	
		vOTU557	*Caudoviricetes*	
		vOTU658	*Caudoviricetes*	
		vOTU675	*Caudoviricetes*	
		vOTU703	*Caudoviricetes*	
		vOTU735	*Caudoviricetes*	
				
GT51	Peptidoglycan glycosyltransferase (EC 2.4.1.129)	vOTU731	*Caudoviricetes*	Genus SYSU-D60009 (*Pseudomonadota*)
				
PL1	Pectate lyase (EC 4.2.2.2,9); Pectin lyase (4.2.2.10)	vOTU361	*Caudoviricetes*	
		vOTU379	*Caudoviricetes*	
		vOTU409	*Caudoviricetes*	

a In addition, the vOTUs with an asterisk contained lysB genes.

## Supplementary Material







## Data Availability

The raw and processed data required to reproduce the above findings are available to download from the Sequence Read Archive (SRA, BioProject number PRJNA1237590).

## Notes

Figure S1. (PDF). Table S1. (DOC). Table S5. (EXCEL) The Supporting Information is available free of charge at https://pubs.acs.org/doi/10.1021/acs.est.5c09194.

HPAEC-PAD chromatograms for compounds identification of biofilm F biomass after total acid hydrolysis, rarefaction curves for the 14 biofilm and water 16S rRNA gene libraries, normalized to the number of reads in the smallest library, evolution of the proportion of different functions detected in the different stages of the biofilms, relative abundances (TAD80/GE) in the three stages of the biofilms of Genomospecies considered as “biofilm producers” or “biofilm degraders”, represented in a heatmap and their abundances of ORFs related to polysaccharides synthesis and degradation shown in a bar graph, relative abundances of the vOTUs in the selected DWDS, which are grouped according to the ICTV taxonomy, distribution of the detected virosphere of the putative hosts, and gene-sharing network of *Mycobacterium*-associated vOTUs and described *Mycobacterium* phages, based on vConTACT2 analysis and graphed with Cytoscape (ZIP)
Main features of the biofilm and water samples used, list of the probes used to determine the distribution of bacterial phyla in biofilm samples with CARD-FISH, physicochemical parameters of the water samples analyzed in this study, and counts of cells/ml and VLP/ml of water samples B, D and F metagenomes characteristics (PDF)
Taxonomic affiliation and abundances of the OTUs obtained from the 14 biofilms and waters 16S rRNA gene libraries, taxonomic affiliation and relative abundances (%) of the genera obtained from the 14 biofilms and waters 16S rRNA gene libraries, taxonomic affiliation and relative abundances of the 16S rRNA genes reads extracted from the metagenomes, functional annotation of contigs above 1kb, genomospecies quality, taxonomy and relative abundances, functional annotation of the genomospecies, and viral OTUs, abundance, taxonomy and host association (ZIP)

## References

[1] Li W., Tan Q., Zhou W., Chen J., Li Y., Wang F., Zhang J. (2020). Impact of substrate material and chlorine/chloramine on the composition
and function of a young biofilm microbial community as revealed by
high-throughput 16S rRNA sequencing. Chemosphere.

[2] Goraj W., Pytlak A., Kowalska B., Kowalski D., Grządziel J., Szafranek-Nakonieczna A., Gałązka A., Stępniewska Z., Stępniewski W. (2021). Influence
of pipe material on biofilm
microbial communities found in drinking water supply system. Environmental Research.

[3] Zhang X., Lin T., Jiang F., Zhang X., Wang S., Zhang S. (2022). Impact of
pipe material and chlorination on the biofilm structure and microbial
communities. Chemosphere.

[4] Campostrini L., Proksch P., Jakwerth S., Farnleitner A. H., Kirschner A. K. T. (2024). Introducing bacterial community turnover
times to elucidate
temporal and spatial hotspots of biological instability in a large
Austrian drinking water distribution network. Water Res..

[5] Learbuch K. L. G., Smidt H., van der Wielen P. W.
J. J. (2022). Water and biofilm
in drinking water distribution systems in the Netherlands. Science of The Total Environment.

[6] Zhang C., Struewing I., Mistry J. H., Wahman D., Pressman J., Lu J. (2021). *Legionella* and other opportunistic pathogens in
full-scale chloraminated municipal drinking water distribution systems. Water Res..

[7] Thom C., Smith C. J., Moore G., Weir P., Ijaz U. Z. (2022). Microbiomes
in drinking water treatment and distribution: A meta-analysis from
source to tap. Water Res..

[8] Zhou X., Zhang K., Zhang T., Li C., Mao X. (2017). An ignored
and potential source of taste and odor (TandO) issues—biofilms
in drinking water distribution system (DWDS). Appl. Microbiol. Biotechnol..

[9] Saxena G., Bharagava R. N., Kaithwas G., Raj A. (2015). Microbial indicators,
pathogens and methods for their monitoring in water environment. Journal of Water and Health.

[10] Oliveira I. M., Gomes I. B., Simões L. C., Simões M. (2024). A review of
research advances on disinfection strategies for biofilm control in
drinking water distribution systems. Water Res..

[11] Brussaard C. P. D. (2004). Optimization
of Procedures for Counting Viruses by Flow Cytometry. Appl. Environ. Microbiol..

[12] Pernthaler A., Pernthaler J., Amann R. (2002). Fluorescence *In Situ* Hybridization and Catalyzed
Reporter Deposition for the Identification
of Marine Bacteria. Appl. Environ. Microbiol..

[13] Escudero C., Vera M., Oggerin M., Amils R. (2018). Active microbial biofilms
in deep poor porous continental subsurface rocks. Scientific Reports.

[14] Pettolino F. A., Walsh C., Fincher G. B., Bacic A. (2012). Determining
the polysaccharide
composition of plant cell walls. Nature Protocols.

[15] Takahashi S., Tomita J., Nishioka K., Hisada T., Nishijima M. (2014). Development
of a prokaryotic universal primer for simultaneous analysis of bacteria
and archaea using next-generation sequencing. PLoS ONE.

[16] Bolyen E., Rideout J. R., Dillon M. R., Bokulich N. A., Abnet C. C., Al-Ghalith G. A., Alexander H., Alm E. J., Arumugam M., Asnicar F., Bai Y., Bisanz J. E., Bittinger K., Brejnrod A., Brislawn C. J., Brown C. T., Callahan B. J., Caraballo-Rodriguez A. M., Chase J., Cope E. K., Da Silva R., Diener C., Dorrestein P. C., Douglas G. M., Durall D. M., Duvallet C., Edwardson C. F., Ernst M., Estaki M., Fouquier J., Gauglitz J. M., Gibbons S. M., Gibson D. L., Gonzalez A., Gorlick K., Guo J., Hillmann B., Holmes S., Holste H., Huttenhower C., Huttley G. A., Janssen S., Jarmusch A. K., Jiang L., Kaehler B. D., Kang K. B., Keefe C. R., Keim P., Kelley S. T., Knights D., Koester I., Kosciolek T., Kreps J., Langille M. G. I., Lee J., Ley R., Liu Y.-X., Loftfield E., Lozupone C., Maher M., Marotz C., Martin B. D., McDonald D., McIver L. J., Melnik A. V., Metcalf J. L., Morgan S. C., Morton J. T., Naimey A. T., Navas-Molina J. A., Nothias L. F., Orchanian S. B., Pearson T., Peoples S. L., Petras D., Preuss M. L., Pruesse E., Rasmussen L. B., Rivers A., Robeson M. S., Rosenthal P., Segata N., Shaffer M., Shiffer A., Sinha R., Song S. J., Spear J. R., Swafford A. D., Thompson L. R., Torres P. J., Trinh P., Tripathi A., Turnbaugh P. J., Ul-Hasan S., van der Hooft J. J. J., Vargas F., Vazquez-Baeza Y., Vogtmann E., von Hippel M., Walters W., Wan Y., Wang M., Warren J., Weber K. C., Williamson C. H. D., Willis A. D., Xu Z. Z., Zaneveld J. R., Zhang Y., Zhu Q., Knight R., Caporaso J. G. (2019). Reproducible, interactive, scalable and extensible
microbiome data science using QIIME 2. Nat.
Biotechnol..

[17] Martin M. (2011). Cutadapt removes
adapter sequences from high-throughput sequencing reads. EMBnet.journal.

[18] Bokulich N. A., Subramanian S., Faith J. J., Gevers D., Gordon J. I., Knight R., Mills D. A., Caporaso J. G. (2013). Quality-filtering
vastly improves diversity estimates from Illumina amplicon sequencing. Nature Methods.

[19] Rognes T., Flouri T., Nichols B., Quince C., Mahé F. (2016). VSEARCH: a
versatile open source tool for metagenomics. PeerJ.

[20] Oksanen A. J., Blanchet F. G., Friendly M., Kindt R., Legendre P., Mcglinn D., Minchin P. R., Hara R. B. O., Simpson G. L., Solymos P., Stevens M. H. H., Szoecs E. (2018). Vegan: Community ecology
package. R package version.

[21] Bokulich N. A., Kaehler B. D., Rideout J. R., Dillon M., Bolyen E., Knight R., Huttley G. A., Gregory Caporaso J. (2018). Optimizing
taxonomic classification of marker-gene amplicon sequences with QIIME
2’s q2-feature-classifier plugin. Microbiome.

[22] Pedregosa F., Varoquaux G., Gramfort A., Michel V., Thirion B., Grisel O., Blondel M., Prettenhofer P., Weiss R., Dubourg V., Vanderplas J., Passos A., Cournapeau D., Brucher M., Perrot M., Duchesnay E. (2011). Scikit-learn:
machine learning in python. Journal of machine
learning research.

[23] Bolger A. M., Lohse M., Usadel B. (2014). Trimmomatic: a flexible trimmer for
Illumina sequence data. Bioinformatics.

[24] Andrews, S. FastQC: a quality control tool for high throughput sequence data, 2010.

[25] Rodriguez-R L. M., Konstantinidis K. T. (2014). Nonpareil:
a redundancy-based approach to assess the
level of coverage in metagenomic datasets. Bioinformatics.

[26] Kopylova E., Noé L., Touzet H. (2012). SortMeRNA: fast and accurate filtering
of ribosomal RNAs in metatranscriptomic data. Bioinformatics.

[27] Quast C., Pruesse E., Yilmaz P., Gerken J., Schweer T., Yarza P., Peplies J., Glöckner F. O. (2012). The SILVA
ribosomal RNA gene database project: improved data processing and
web-based tools. Nucleic Acids Res..

[28] Rodriguez-R L. M., Konstantinidis K. T. (2016). The enveomics
collection: a toolbox for specialized
analyses of microbial genomes and metagenomes. PeerJ..

[29] Konstantinidis K. T., Rosselló-Móra R., Amann R. (2017). Uncultivated microbes
in need of their own taxonomy. The ISME Journal.

[30] Peng Y., Leung H. C. M., Yiu S. M., Chin F. Y. L. (2012). IDBA-UD: a de
novo assembler for single-cell and metagenomic sequencing data with
highly uneven depth. Bioinformatics.

[31] Nurk S., Meleshko D., Korobeynikov A., Pevzner P. A. (2017). metaSPAdes: a new
versatile metagenomic assembler. Genome Res..

[32] Li D., Liu C.-M., Luo R., Sadakane K., Lam T.-W. (2015). MEGAHIT:
an ultra-fast single-node solution for large and complex metagenomics
assembly via succinct de Bruijn graph. Bioinformatics.

[33] Hyatt D., Chen G.-L., LoCascio P. F., Land M. L., Larimer F. W., Hauser L. J. (2010). Prodigal: prokaryotic gene recognition and translation
initiation site identification. BMC Bioinformatics.

[34] Eren A. M., Kiefl E., Shaiber A., Veseli I., Miller S. E., Schechter M. S., Fink I., Pan J. N., Yousef M., Fogarty E. C., Trigodet F., Watson A. R., Esen O. C., Moore R. M., Clayssen Q., Lee M. D., Kivenson V., Graham E. D., Merrill B. D., Karkman A., Blankenberg D., Eppley J. M., Sjodin A., Scott J. J., Vazquez-Campos X., McKay L. J., McDaniel E. A., Stevens S. L. R., Anderson R. E., Fuessel J., Fernandez-Guerra A., Maignien L., Delmont T. O., Willis A. D. (2020). Community-led, integrated, reproducible multi-omics
with anvi’o. Nature Microbiology.

[35] Kanehisa M., Goto S. (2000). KEGG: Kyoto encyclopedia of genes and genomes. Nucleic Acids Res..

[36] Kanehisa M., Furumichi M., Tanabe M., Sato Y., Morishima K. (2017). KEGG: new
perspectives on genomes, pathways, diseases and drugs. Nucleic Acids Res..

[37] Zheng J., Ge Q., Yan Y., Zhang X., Huang L., Yin Y. (2023). dbCAN3: automated
carbohydrate-active enzyme and substrate annotation. Nucleic Acids Res..

[38] Drula E., Garron M.-L., Dogan S., Lombard V., Henrissat B., Terrapon N. (2022). The carbohydrate-active enzyme database: functions
and literature. Nucleic Acids Res..

[39] Zhang H., Yohe T., Huang L., Entwistle S., Wu P., Yang Z., Busk P. K., Xu Y., Yin Y. (2018). dbCAN2: a
meta server for automated carbohydrate-active enzyme annotation. Nucleic Acids Res..

[40] Marrakchi H., Lanéelle M.-A., Daffé M. (2014). Mycolic Acids: Structures, Biosynthesis,
and Beyond. Chemistry and Biology.

[41] Dueholm M. K. D., Besteman M., Zeuner E. J., Riisgaard-Jensen M., Nielsen M. E., Vestergaard S. Z., Heidelbach S., Bekker N. S., Nielsen P. H. (2023). Genetic potential
for exopolysaccharide
synthesis in activated sludge bacteria uncovered by genome-resolved
metagenomics. Water Res..

[42] Bairoch A. (2000). The ENZYME
database in 2000. Nucleic Acids Res..

[43] Boutet E., Lieberherr D., Tognolli M., Schneider M., Bairoch A., Edwards D. (2007). UniProtKB/Swiss-Prot. Methods in Molecular Biology.

[44] McArthur A. G., Waglechner N., Nizam F., Yan A., Azad M. A., Baylay A. J., Bhullar K., Canova M. J., De Pascale G., Ejim L., Kalan L., King A. M., Koteva K., Morar M., Mulvey M. R., O’Brien J. S., Pawlowski A. C., Piddock L. J. V., Spanogiannopoulos P., Sutherland A. D., Tang I., Taylor P. L., Thaker M., Wang W., Yan M., Yu T., Wright G. D. (2013). The Comprehensive
Antibiotic Resistance Database. Antimicrobial
Agents and Chemotherapy.

[45] Alcock B. P., Raphenya A. R., Lau T. T. Y., Tsang K. K., Bouchard M., Edalatmand A., Huynh W., Nguyen A.-L. V., Cheng A. A., Liu S., Min S. Y., Miroshnichenko A., Tran H.-K., Werfalli R. E., Nasir J. A., Oloni M., Speicher D. J., Florescu A., Singh B., Faltyn M., Hernandez-Koutoucheva A., Sharma A. N., Bordeleau E., Pawlowski A. C., Zubyk H. L., Dooley D., Griffiths E., Maguire F., Winsor G. L., Beiko R. G., Brinkman F. S. L., Hsiao W. W. L., Domselaar G. V., McArthur A. G. (2019). CARD 2020: antibiotic
resistome surveillance with the comprehensive antibiotic resistance
database. Nucleic Acids Res..

[46] Díaz-Rullo J., González-Pastor J. E. (2023). tRNA queuosine modification is involved
in biofilm formation and virulence in bacteria. Nucleic Acids Res..

[47] Wu Y.-W., Simmons B. A., Singer S. W. (2016). MaxBin
2.0: an automated binning
algorithm to recover genomes from multiple metagenomic datasets. Bioinformatics.

[48] Kang D. D., Li F., Kirton E., Thomas A., Egan R., An H., Wang Z. (2019). MetaBAT 2:
an adaptive binning algorithm for robust and efficient
genome reconstruction from metagenome assemblies. PeerJ.

[49] Chklovski A., Parks D. H., Woodcroft B. J., Tyson G. W. (2023). CheckM2: a rapid,
scalable and accurate tool for assessing microbial genome quality
using machine learning. Nature Methods..

[50] Sieber C. M. K., Probst A. J., Sharrar A., Thomas B. C., Hess M., Tringe S. G., Banfield J. F. (2018). Recovery
of genomes from metagenomes
via a dereplication, aggregation and scoring strategy. Nature Microbiology.

[51] Orellana L. H., Francis T. B., Ferraro M., Hehemann J.-H., Fuchs B. M., Amann R. I. (2022). *Verrucomicrobiota* are specialist consumers
of sulfated methyl pentoses during diatom blooms. The ISME Journal..

[52] Sayers E. W., Bolton E. E., Brister J. R., Canese K., Chan J., Comeau D. C., Connor R., Funk K., Kelly C., Kim S., Madej T., Marchler-Bauer A., Lanczycki C., Lathrop S., Lu Z., Thibaud-Nissen F., Murphy T., Phan L., Skripchenko Y., Tse T., Wang J., Williams R., Trawick B. W., Pruitt K. D., Sherry S. T. (2022). Database resources of the national center for biotechnology
information. Nucleic Acids Res..

[53] Ramos-Barbero M. D., Martin-Cuadrado A.-B., Viver T., Santos F., Martinez-Garcia M., Antón J. (2019). Recovering microbial genomes from metagenomes in hypersaline
environments: The Good, the Bad and the Ugly. Systematic and Applied Microbiology.

[54] Rodriguez-R L. M., Conrad R. E., Viver T., Feistel D. J., Lindner B. G., Venter S. N., Orellana L. H., Amann R., Rossello-Mora R., Konstantinidis K. T. (2024). An ANI
gap within bacterial species that advances the
definitions of intra-species units. mBio..

[55] Olm M. R., Brown C. T., Brooks B., Banfield J. F. (2017). dRep: a tool for
fast and accurate genomic comparisons that enables improved genome
recovery from metagenomes through de-replication. The ISME Journal.

[56] Li H., Handsaker B., Wysoker A., Fennell T., Ruan J., Homer N., Marth G., Abecasis G., Durbin R. (2009). The Sequence
Alignment/Map format and SAMtools. Bioinformatics.

[57] Castro J. C., Rodriguez-R L. M., Harvey W. T., Weigand M. R., Hatt J. K., Carter M. Q., Konstantinidis K. T. (2018). imGLAD: accurate detection and quantification
of target organisms in metagenomes. PeerJ.

[58] Lindner B. G., Gerhardt K., Feistel D. J., Rodriguez-R L. M., Hatt J. K., Konstantinidis K. T. (2024). A user’s
guide to the bioinformatic
analysis of shotgun metagenomic sequence data for bacterial pathogen
detection. Int. J. Food Microbiol..

[59] Quinlan A. R., Hall I. M. (2010). BEDTools: a flexible
suite of utilities for comparing
genomic features. Bioinformatics.

[60] Langmead B., Salzberg S. L. (2012). Fast gapped-read
alignment with Bowtie 2. Nature Methods.

[61] Nayfach S., Pollard K. S. (2015). Average genome size
estimation improves comparative
metagenomics and sheds light on the functional ecology of the human
microbiome. Genome Biology.

[62] Chaumeil P.-A., Mussig A. J., Hugenholtz P., Parks D. H. (2022). GTDB-Tk v2: memory
friendly classification with the Genome Taxonomy Database. Bioinformatics..

[63] Asnicar F., Thomas A. M., Beghini F., Mengoni C., Manara S., Manghi P., Zhu Q., Bolzan M., Cumbo F., May U., Sanders J. G., Zolfo M., Kopylova E., Pasolli E., Knight R., Mirarab S., Huttenhower C., Segata N. (2020). Precise phylogenetic analysis of microbial isolates
and genomes from metagenomes using PhyloPhlAn 3.0. Nature Communications.

[64] Letunic I., Bork P. (2021). Interactive Tree Of Life (iTOL) v5:
an online tool for phylogenetic
tree display and annotation. Nucleic Acids Res..

[65] Couvin D., Bernheim A., Toffano-Nioche C., Touchon M., Michalik J., Néron B., Rocha E. P. C., Vergnaud G., Gautheret D., Pourcel C. (2018). CRISPRCasFinder, an update of CRISRFinder, includes
a portable version, enhanced performance and integrates search for
Cas proteins. Nucleic Acids Res..

[66] Kieft K., Zhou Z., Anantharaman K. (2020). VIBRANT: automated
recovery, annotation
and curation of microbial viruses, and evaluation of viral community
function from genomic sequences. Microbiome.

[67] Guo J., Bolduc B., Zayed A. A., Varsani A., Dominguez-Huerta G., Delmont T. O., Pratama A. A., Gazitúa M. C., Vik D., Sullivan M. B., Roux S. (2021). VirSorter2:
a multi-classifier, expert-guided
approach to detect diverse DNA and RNA viruses. Microbiome.

[68] Nayfach S., Camargo A. P., Schulz F., Eloe-Fadrosh E., Roux S., Kyrpides N. C. (2021). CheckV assesses the quality and completeness
of metagenome-assembled viral genomes. Nat.
Biotechnol..

[69] Ramos-Barbero M. D., Viver T., Zabaleta A., Senel E., Gomariz M., Antigüedad I., Santos F., Martínez-García M., Rosselló-Móra R., Antón J. (2021). Ancient saltern
metagenomics: tracking changes in microbes and their viruses from
the underground to the surface. Environmental
Microbiology.

[70] Nayfach S., Páez-Espino D., Call L., Low S. J., Sberro H., Ivanova N. N., Proal A. D., Fischbach M. A., Bhatt A. S., Hugenholtz P., Kyrpides N. C. (2021). Metagenomic compendium
of 189,680 DNA viruses from the human gut microbiome. Nature Microbiology.

[71] Camargo A. P., Roux S., Schulz F., Babinski M., Xu Y., Hu B., Chain P. S. G., Nayfach S., Kyrpides N. C. (2024). Identification of
mobile genetic elements with geNomad. Nat. Biotechnol..

[72] Guajardo-Leiva S., Santos F., Salgado O., Regeard C., Quillet L., Díez B. (2021). Unveiling
ecological and genetic novelty within lytic
and lysogenic viral communities of hot spring phototrophic microbial
mats. Microbiology Spectrum.

[73] Roux S., Camargo A. P., Coutinho F. H., Dabdoub S. M., Dutilh B. E., Nayfach S., Tritt A. (2023). iPHoP: An
integrated machine learning
framework to maximize host prediction for metagenome-derived viruses
of archaea and bacteria. PLOS Biology.

[74] Bin
Jang H., Bolduc B., Zablocki O., Kuhn J. H., Roux S., Adriaenssens E. M., Brister J. R., Kropinski A. M., Krupovic M., Lavigne R., Turner D., Sullivan M. B. (2019). Taxonomic
assignment of uncultivated prokaryotic virus genomes is enabled by
gene-sharing networks. Nat. Biotechnol..

[75] Otasek D., Morris J. H., Bouças J., Pico A. R., Demchak B. (2019). Cytoscape
Automation: empowering workflow-based network analysis. Genome Biology.

[76] Anderson M. J. (2001). A new method
for non-parametric multivariate analysis of variance. Austral Ecology.

[77] Wickham, H. ggplot2; Springer International Publishing: 2016. 10.1007/978-3-319-24277-4.

[78] Pan S., Underhill S. A. M., Hamm C. W., Stover M. A., Butler D. R., Shults C. A., Manjarrez J. R., Cabeen M. T. (2024). Glycerol metabolism
impacts biofilm phenotypes and virulence in *Pseudomonas aeruginosa* via the Entner-Doudoroff pathway. MSphere..

[79] Good I. J. (1953). The population
frequencies of species and the estimation of population parameters. Biometrika.

[80] Thomas R. Z., Zijnge V., Ciçek A., de Soet J. J., Harmsen H. J. M., Huysmans M. C. D. N. J. M. (2012). Shifts
in the microbial population
in relation to in situ caries progression. Caries
Research.

[81] Romaní A. M., Borrego C. M., Díaz-Villanueva V., Freixa A., Gich F., Ylla I. (2014). Shifts in microbial community structure
and function in light- and dark-grown biofilms driven by warming. Environmental Microbiology.

[82] Cheng Q., Liu Z., Huang Y., Li F., Nengzi L., Zhang J. (2020). Influence
of temperature on CODMn and Mn2+ removal and microbial community structure
in pilot-scale biofilter. Bioresour. Technol..

[83] Kim I., Chhetri G., Kim J., So Y., Seo T. (2023). *Chryseolinea
lacunae* sp. nov. *and piscinibacter lacus* sp. nov. isolated from artificial pond water. Curr. Microbiol..

[84] Pedrós-Alió C. (2006). Marine microbial
diversity: can it be determined?. Trends in
Microbiology.

[85] Haig S.-J., Kotlarz N., LiPuma J. J., Raskin L. (2018). A high-throughput
approach
for identification of nontuberculous mycobacteria in drinking water
reveals relationship between water age and *Mycobacterium avium*. MBio.

[86] Zhu J., Liu R., Cao N., Yu J., Liu X., Yu Z. (2019). Mycobacterial
metabolic characteristics in a water meter biofilm revealed by metagenomics
and metatranscriptomics. Water Res..

[87] Perrin Y., Bouchon D., Delafont V., Moulin L., Héchard Y. (2019). Microbiome
of drinking water: A full-scale spatio-temporal study to monitor water
quality in the Paris distribution system. Water
Res..

[88] Calero
Preciado C., Husband S., Boxall J., del Olmo G., Soria-Carrasco V., Maeng S. K., Douterelo I. (2021). Intermittent
water supply impacts on distribution system biofilms and water quality. Water Res..

[89] Vaz-Moreira I., Nunes O. C., Manaia C. M. (2011). Diversity and antibiotic
resistance
patterns of *Sphingomonadaceae* isolates from drinking
water. Appl. Environ. Microbiol..

[90] Gulati P., Ghosh M. (2017). Biofilm forming ability of *Sphingomonas paucimobilis* isolated from community drinking water systems on plumbing materials
used in water distribution. Journal of Water
and Health.

[91] Kalmbach S. (2000). In situ probing
reveals *Aquabacterium* commune as a widespread and
highly abundant bacterial species in drinking water biofilms. Water Res..

[92] Gheorghita A. A., Wozniak D. J., Parsek M. R., Howell P. L. (2023). *Pseudomonas
aeruginosa* biofilm exopolysaccharides: Assembly, function,
and degradation. FEMS Microbiology Reviews..

[93] Sala-Comorera L., Caudet-Segarra L., Galofré B., Lucena F., Blanch A. R., García-Aljaro C. (2020). Unravelling
the composition of tap and mineral water
microbiota: Divergences between next-generation sequencing techniques
and culture-based methods. Int. J. Food Microbiol..

[94] Im H. R., Im S. J., Nguyen D. V., Jeong S. P., Jang A. (2024). Real-time
diagnosis and monitoring of biofilm and corrosion layer formation
on different water pipe materials using non-invasive imaging methods. Chemosphere.

[95] Kanehisa M. (2004). The KEGG resource
for deciphering the genome. Nucleic Acids Res..

[96] Lombard V., Golaconda Ramulu H., Drula E., Coutinho P. M., Henrissat B. (2014). The carbohydrate-active
enzymes database (CAZy) in 2013. Nucleic Acids
Res..

[97] Kragh K. N., Tolker-Nielsen T., Lichtenberg M. (2023). The non-attached biofilm aggregate. Communications Biology.

[98] Sauer K., Stoodley P., Goeres D. M., Hall-Stoodley L., Burmølle M., Stewart P. S., Bjarnsholt T. (2022). The biofilm
life cycle: expanding the conceptual model of biofilm formation. Nature Reviews Microbiology..

[99] Ojha A. K., Baughn A. D., Sambandan D., Hsu T., Trivelli X., Guerardel Y., Alahari A., Kremer L., Jacobs W. R., Hatfull G. F. (2008). Growth of *Mycobacterium tuberculosis* biofilms containing free mycolic acids and harbouring drug-tolerant
bacteria. Mol. Microbiol..

[100] Ito A., Taniuchi A., May T., Kawata K., Okabe S. (2009). Increased
antibiotic resistance of escherichia coli in mature biofilms. Appl. Environ. Microbiol..

[101] Zack K. M., Sorenson T., Joshi S. G. (2024). Types and
mechanisms
of efflux pump systems and the potential of efflux pump inhibitors
in the restoration of antimicrobial susceptibility, with a special
reference to *Acinetobacter baumannii*. Pathogens.

[102] Coyne S., Rosenfeld N., Lambert T., Courvalin P., Périchon B. (2010). Overexpression of Resistance-Nodulation-Cell Division
Pump AdeFGH Confers Multidrug Resistance in *Acinetobacter
baumannii*. Antimicrobial Agents and
Chemotherapy.

[103] Girlich D., Naas T., Nordmann P. (2004). Biochemical characterization
of the naturally occurring oxacillinase OXA-50 of *pseudomonas
aeruginosa*. Antimicrobial Agents and
Chemotherapy.

[104] Park A. K., Kim H., Jin H. J. (2010). Phylogenetic
analysis
of rRNA methyltransferases, Erm and KsgA, as related to antibiotic
resistance. FEMS Microbiology Letters.

[105] Prasertanan T., Palmer D. R. J. (2019). The kanosamine
biosynthetic pathway
in *Bacillus cereus* UW85: Functional and kinetic characterization
of KabA, KabB, and KabC. Arch. Biochem. Biophys..

[106] Pawar S., Chaudhari A., Prabha R., Shukla R., Singh D. P. (2019). Microbial Pyrrolnitrin:
Natural Metabolite with Immense
Practical Utility. Biomolecules.

[107] Dey A., Verma A. K., Chatterji D. (2011). Molecular
insights into the mechanism
of phenotypic tolerance to rifampicin conferred on mycobacterial RNA
polymerase by MsRbpA. Microbiology.

[108] De Smet K. A. L., Kempsell K. E., Gallagher A., Duncan K., Young D. B. (1999). Alteration
of a single amino acid
residue reverses fosfomycin resistance of recombinant MurA from *Mycobacterium tuberculosis*. Microbiology.

[109] Miao X., Han X., Liu C., Bai X. (2022). Intrinsic
chlorine resistance of bacteria modulated by glutaminyl-tRNA biosynthesis
in drinking water supply systems. Chemosphere.

[110] Pereira-Garcia C., Sanz-Sáez I., Sánchez P., Coutinho F. H., Bravo A. G., Sánchez O., Acinas S. G. (2024). Genomic and transcriptomic characterization of methylmercury
detoxification in a deep ocean *Alteromonas mediterranea* ISS312. Environ. Pollut..

[111] Abtahi M., Dobaradaran S., Torabbeigi M., Jorfi S., Gholamnia R., Koolivand A., Darabi H., Kavousi A., Saeedi R. (2019). Health risk
of phthalates
in water environment: Occurrence in water resources, bottled water,
and tap water, and burden of disease from exposure through drinking
water in tehran, Iran. Environmental Research.

[112] Ma X., Deng J., Feng J., Shanaiah N., Smiley E., Dietrich A. M. (2016). Identification and
characterization of phenylacetonitrile
as a nitrogenous disinfection byproduct derived from chlorination
of phenylalanine in drinking water. Water Res..

[113] McKee L. S., La Rosa S. L., Westereng B., Eijsink V. G., Pope P. B., Larsbrink J. (2021). Polysaccharide
degradation by the Bacteroidetes: Mechanisms and nomenclature. Environmental Microbiology Reports.

[114] Wiegand S., Jogler M., Jogler C. (2018). On the maverick
Planctomycetes. FEMS Microbiology Reviews.

[115] Azeredo J., García P., Drulis-Kawa Z. (2021). Targeting
biofilms using phages and their enzymes. Current
Opinion in Biotechnology.

[116] Hegarty B., Dai Z., Raskin L., Pinto A., Wigginton K., Duhaime M. (2022). A snapshot of the global drinking
water virome: diversity and metabolic potential vary with residual
disinfectant use. Water Res..

[117] Hatfull G. F. (2018). Mycobacteriophages. Microbiology
Spectrum.

[118] Payne K., Sun Q., Sacchettini J., Hatfull G. F. (2009). Mycobacteriophage Lysin B is a novel
mycolylarabinogalactan
esterase. Mol. Microbiol..

[119] Singh A. K., Gangakhedkar R., Thakur H. S., Raman S. K., Patil S. A., Jain V. (2023). Mycobacteriophage
D29 Lysin B exhibits
promising anti-mycobacterial activity against drug-resistant *Mycobacterium tuberculosis*. Microbiology
Spectrum..

