# Supplementary material for: Microbial Biofilms Dynamics and Functionality in an Urban Mycobacterium-Dominated Drinking Water Distribution System
Source: Environ Sci Technol. 2026 Feb 9;60(7):5242–58. doi: 10.1021/acs.est.5c09194 (PMC12947685; doi:10.1021/acs.est.5c09194)
Supplement: Supplementary file 1 [file es5c09194_si_001.zip › Gangloff et al 2025_suppl_figures_corrected.pptx]

## Slide 1
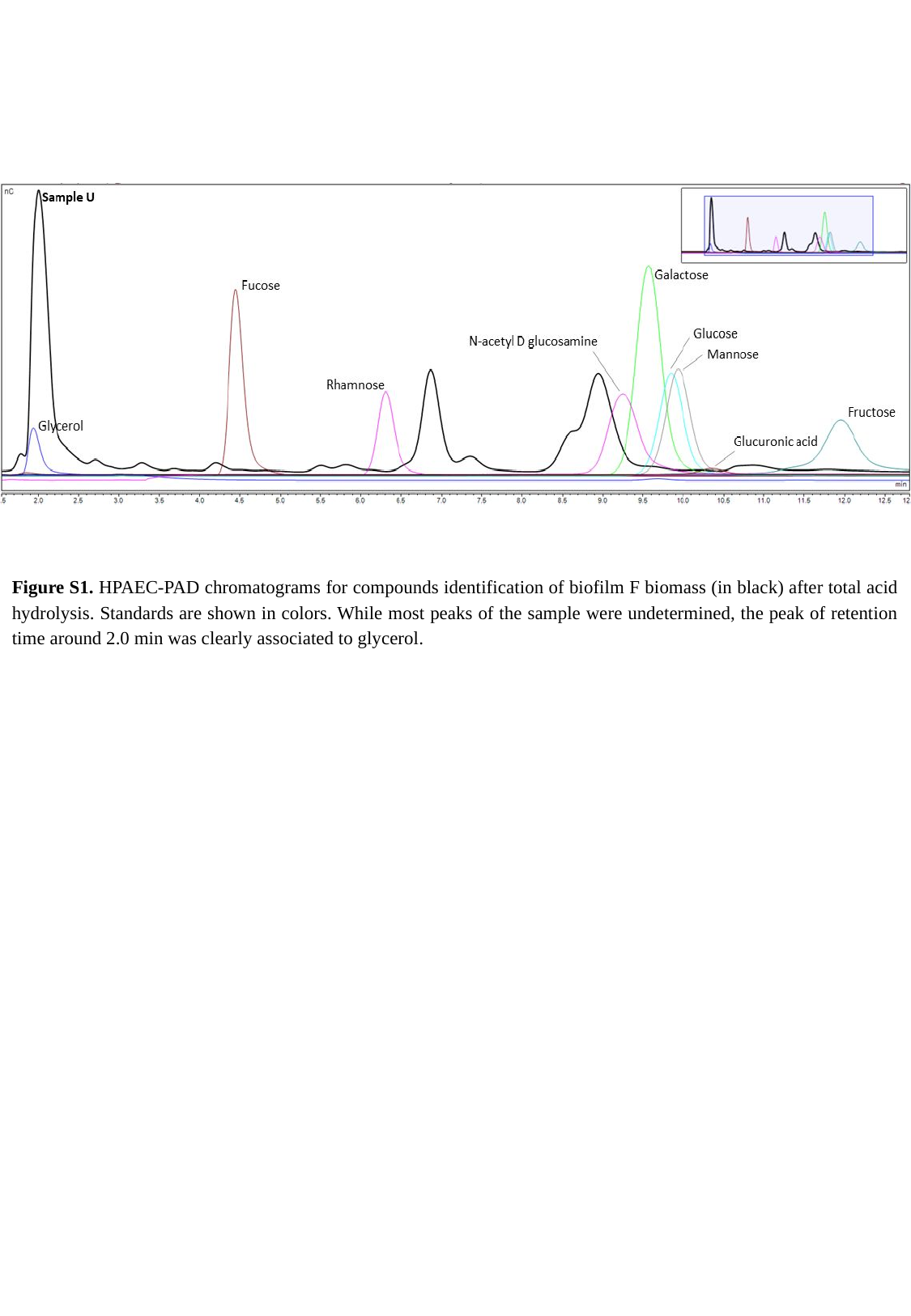

Figure S1. HPAEC-PAD chromatograms for compounds identification of biofilm F biomass (in black) after total acid hydrolysis. Standards are shown in colors. While most peaks of the sample were undetermined, the peak of retention time around 2.0 min was clearly associated to glycerol.

## Slide 2
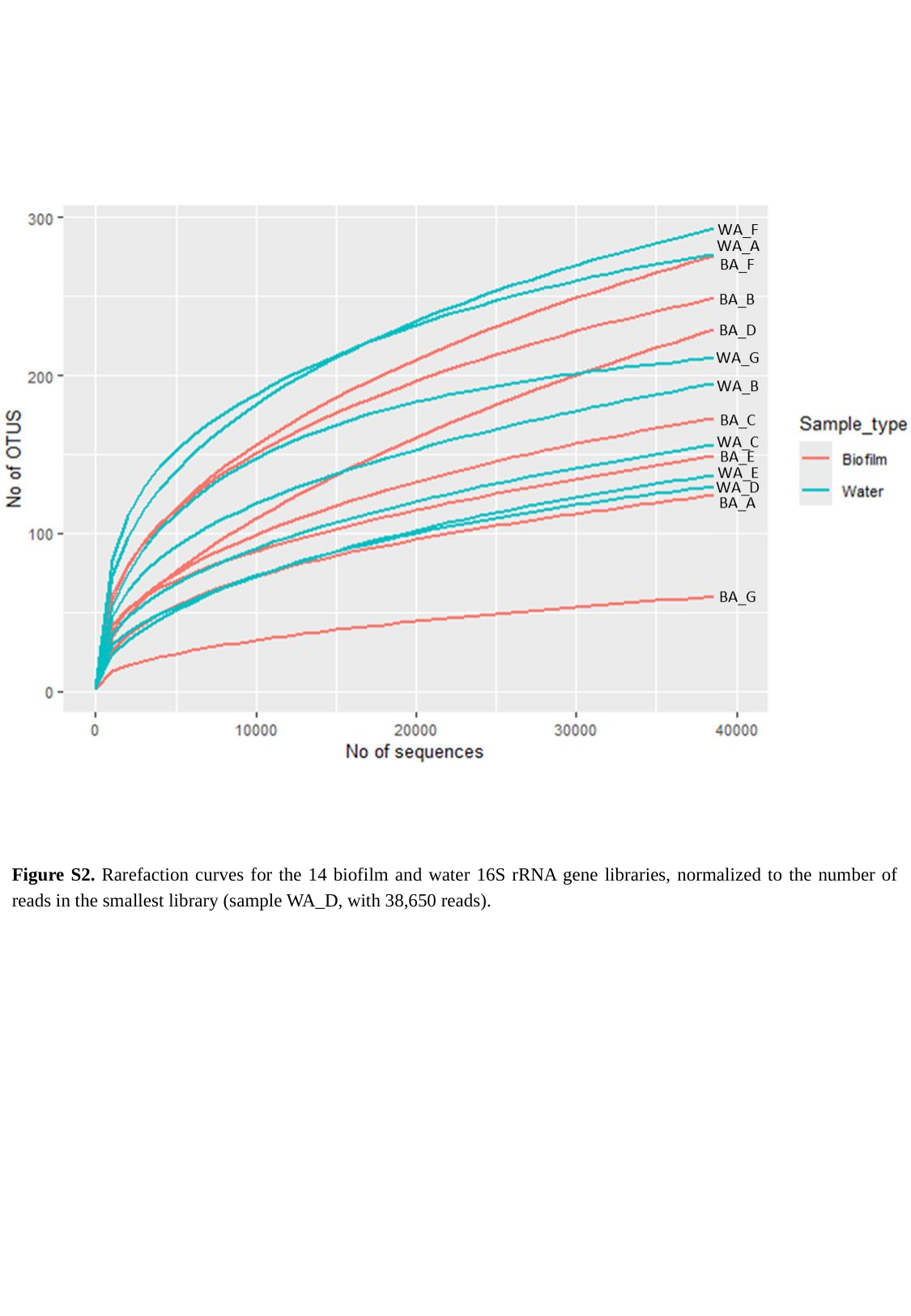

Figure S2. Rarefaction curves for the 14 biofilm and water 16S rRNA gene libraries, normalized to the number of reads in the smallest library (sample WA_D, with 38,650 reads).

## Slide 3
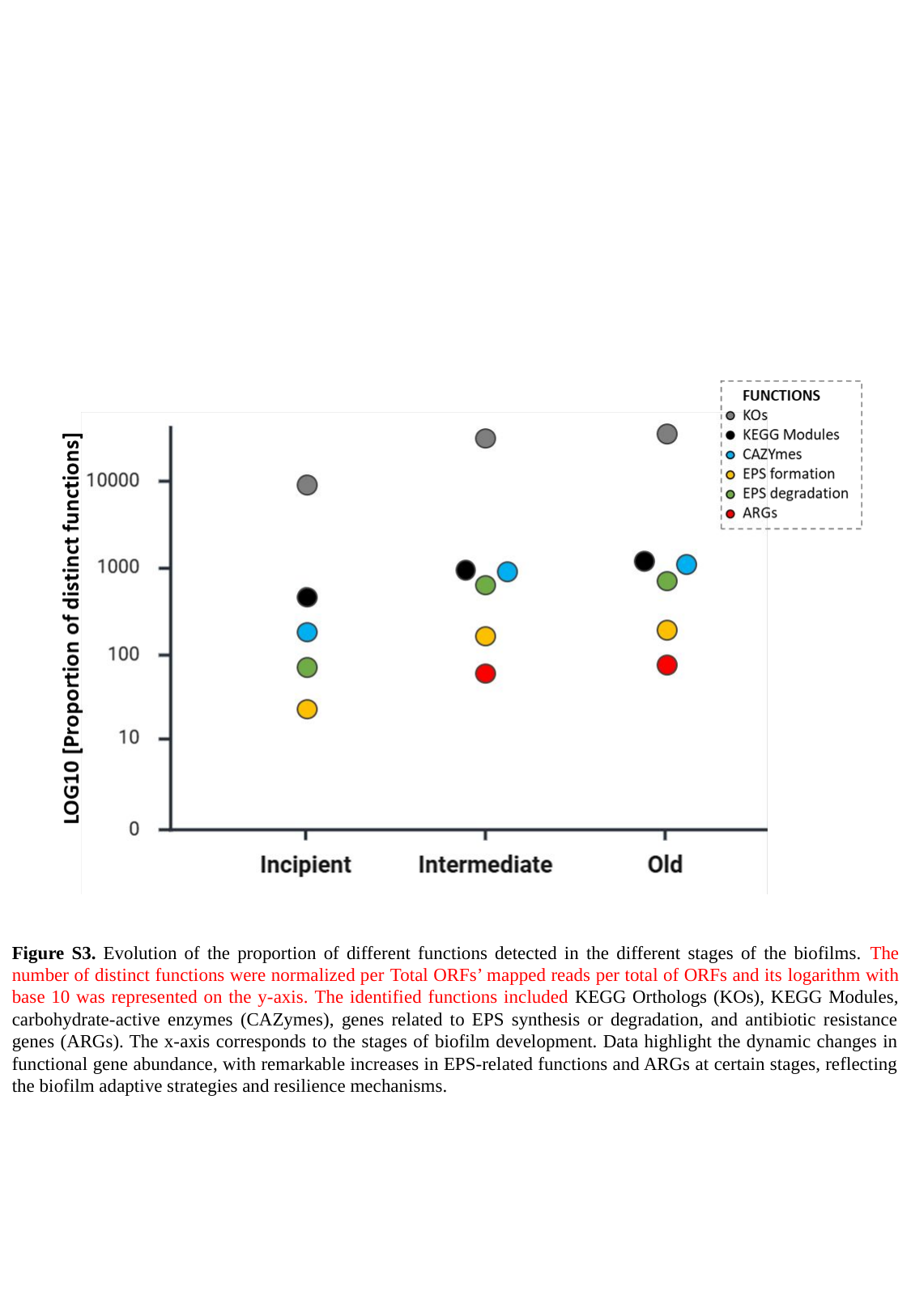

Figure S3. Evolution of the proportion of different functions detected in the different stages of the biofilms. The number of distinct functions were normalized per Total ORFs’ mapped reads per total of ORFs and its logarithm with base 10 was represented on the y-axis. The identified functions included KEGG Orthologs (KOs), KEGG Modules, carbohydrate-active enzymes (CAZymes), genes related to EPS synthesis or degradation, and antibiotic resistance genes (ARGs). The x-axis corresponds to the stages of biofilm development. Data highlight the dynamic changes in functional gene abundance, with remarkable increases in EPS-related functions and ARGs at certain stages, reflecting the biofilm adaptive strategies and resilience mechanisms.

## Slide 4
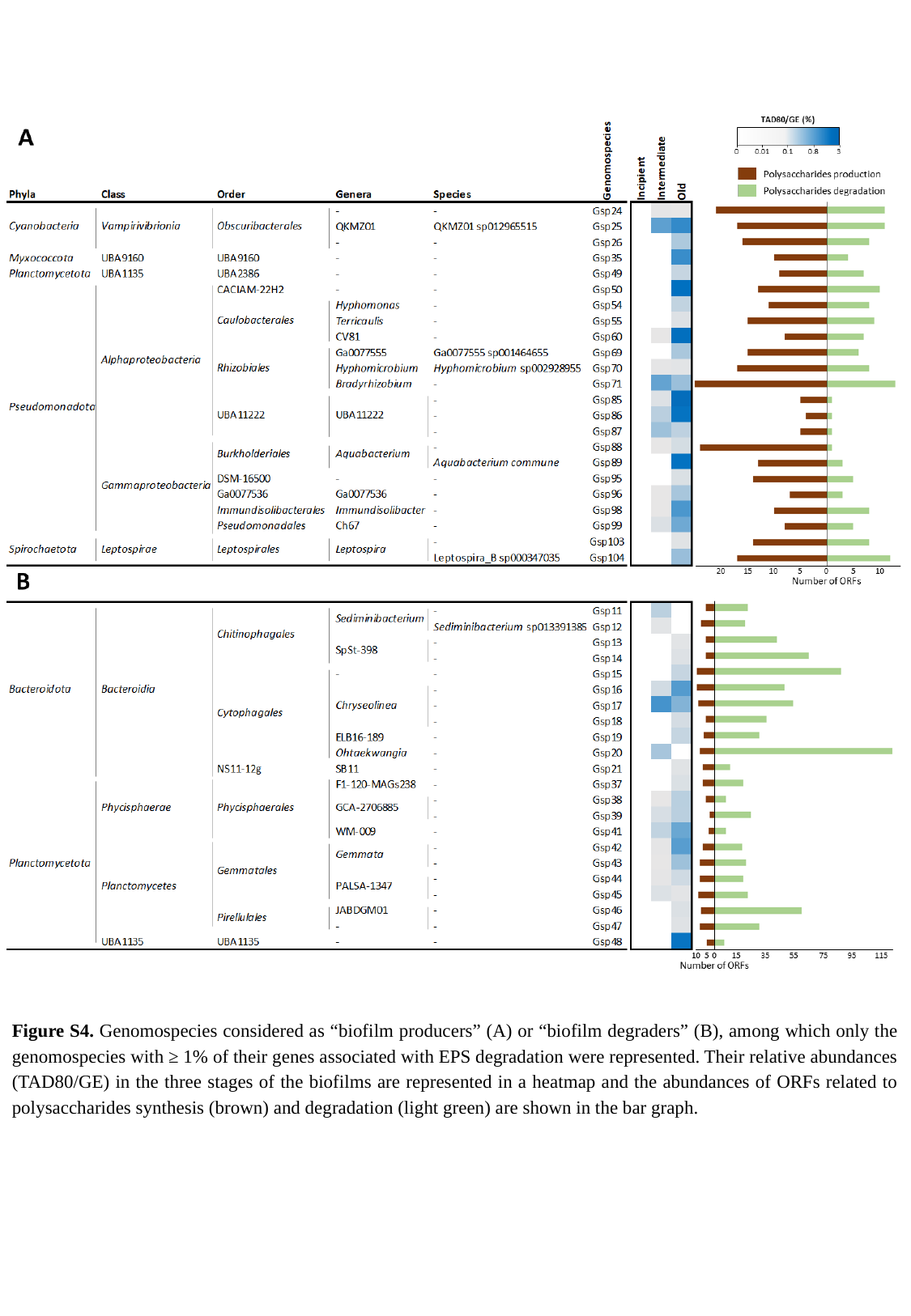

Figure S4. Genomospecies considered as “biofilm producers” (A) or “biofilm degraders” (B), among which only the genomospecies with ≥ 1% of their genes associated with EPS degradation were represented. Their relative abundances (TAD80/GE) in the three stages of the biofilms are represented in a heatmap and the abundances of ORFs related to polysaccharides synthesis (brown) and degradation (light green) are shown in the bar graph.

## Slide 5
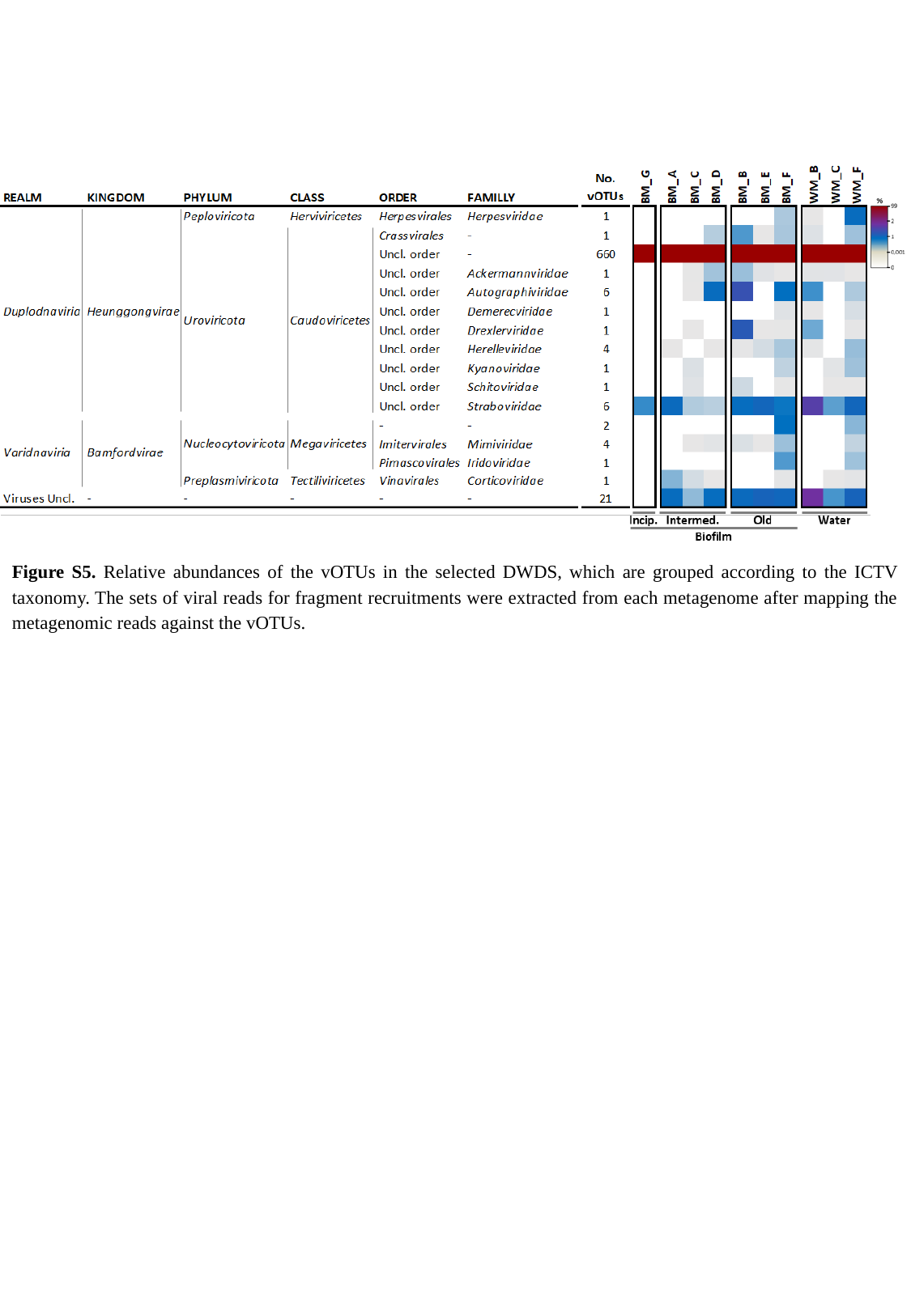

Figure S5. Relative abundances of the vOTUs in the selected DWDS, which are grouped according to the ICTV taxonomy. The sets of viral reads for fragment recruitments were extracted from each metagenome after mapping the metagenomic reads against the vOTUs.

## Slide 6
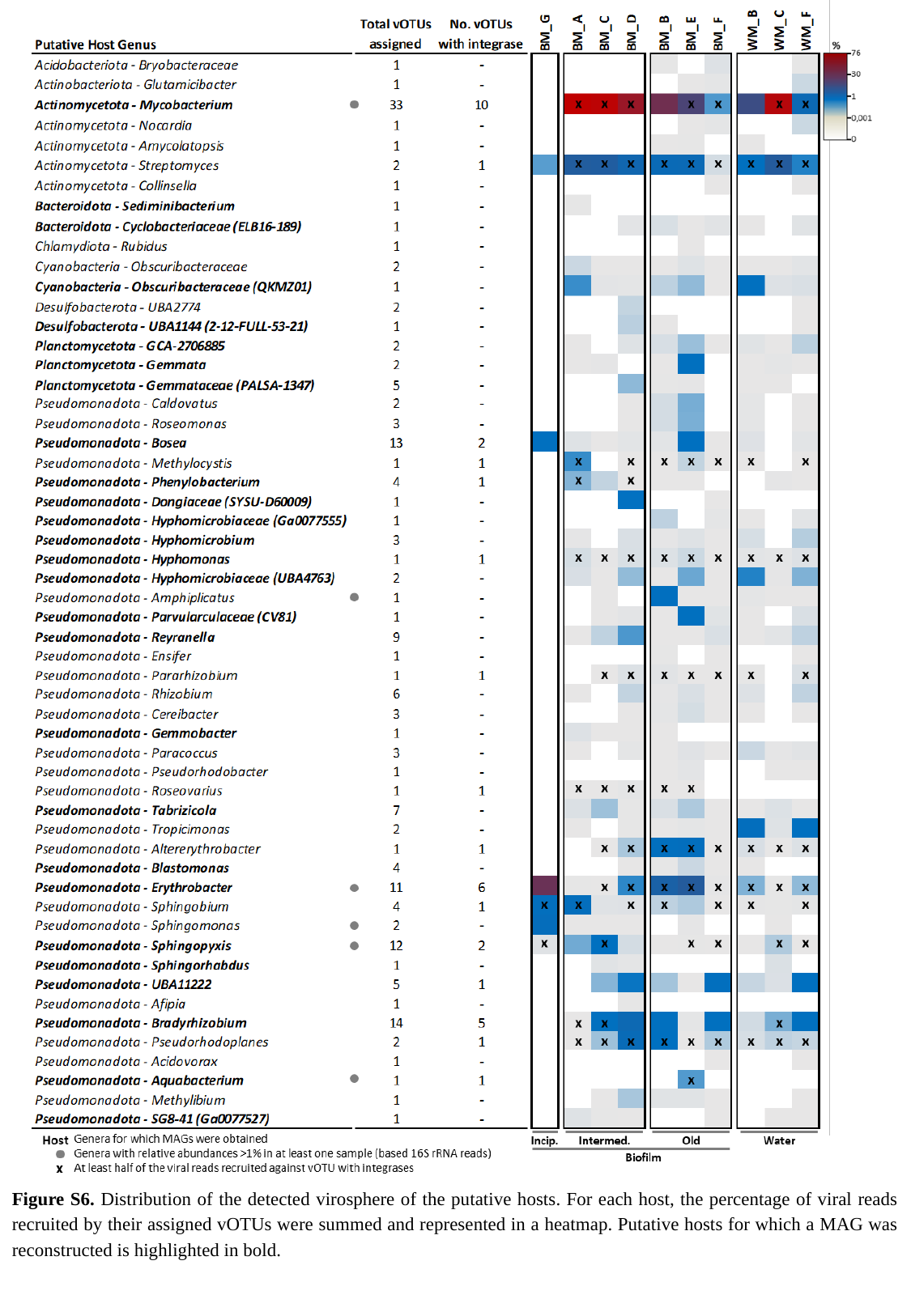

Figure S6. Distribution of the detected virosphere of the putative hosts. For each host, the percentage of viral reads recruited by their assigned vOTUs were summed and represented in a heatmap. Putative hosts for which a MAG was reconstructed is highlighted in bold.

## Slide 7
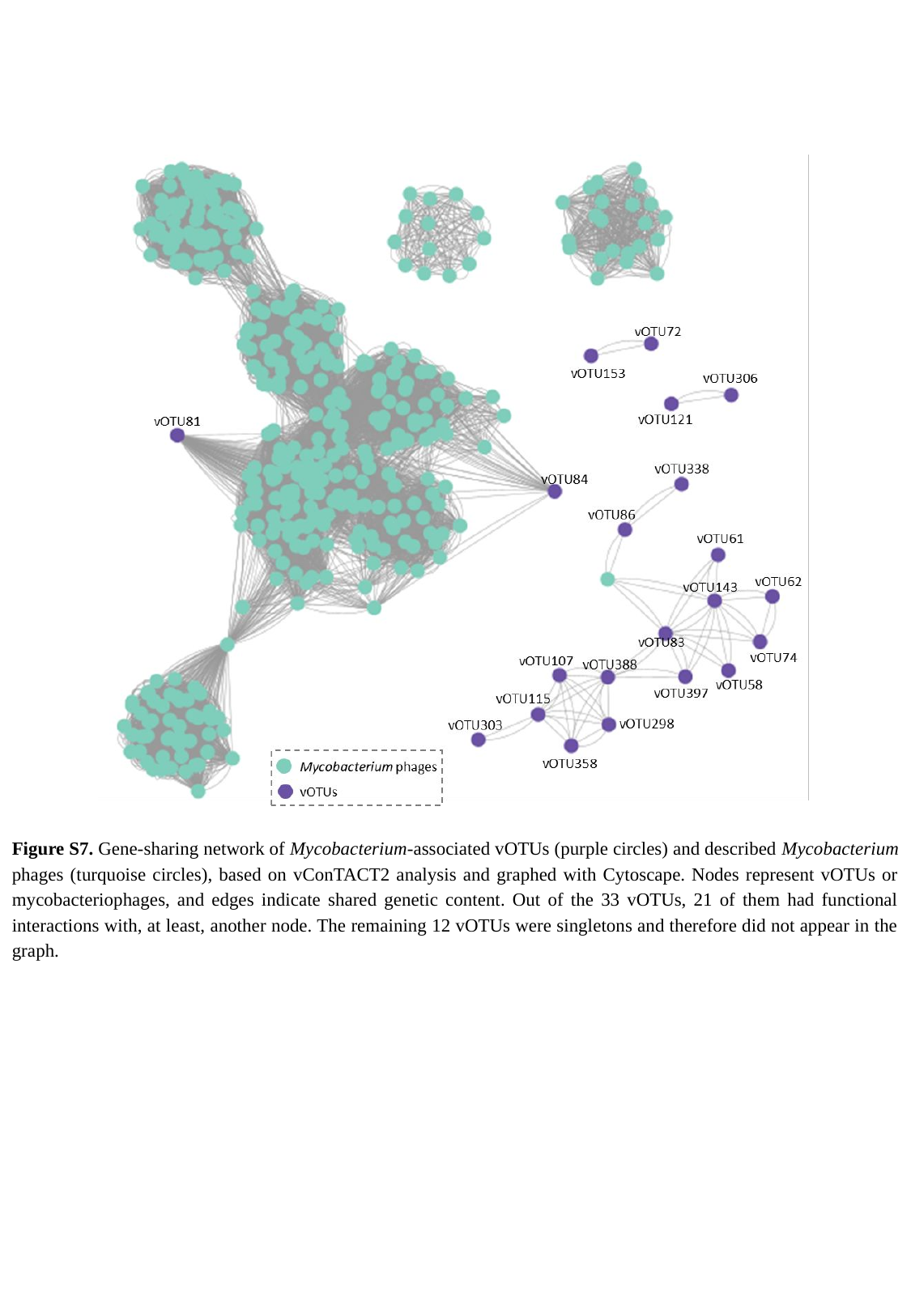

Figure S7. Gene-sharing network of Mycobacterium-associated vOTUs (purple circles) and described Mycobacterium phages (turquoise circles), based on vConTACT2 analysis and graphed with Cytoscape. Nodes represent vOTUs or mycobacteriophages, and edges indicate shared genetic content. Out of the 33 vOTUs, 21 of them had functional interactions with, at least, another node. The remaining 12 vOTUs were singletons and therefore did not appear in the graph.
