# Supplementary material for: Microbial Biofilms Dynamics and Functionality in an Urban Mycobacterium-Dominated Drinking Water Distribution System
Source: Environ Sci Technol. 2026 Feb 9;60(7):5242–58. doi: 10.1021/acs.est.5c09194 (PMC12947685; doi:10.1021/acs.est.5c09194)
Supplement: Supplementary file 2 [file es5c09194_si_002.pdf]

A manuscript submitted to *Environmental Science & Technology*

Microbial biofilms dynamics and functionality in an urban *Mycobacterium*-dominated Drinking Water Distribution System

Valentin Gangloff[1], Borja Aldeguer-Riquelme[1,2], Mª Adela Yañez[3], Gabrielle Potocki-Veronese[4], Etienne Severac[4], Josefa Antón[1,5,6], Elena Soria [1, 3] and Fernando Santos [1,6*]

[1] Department of Physiology, Genetics, and Microbiology, University of Alicante, Alicante, 03080, Spain

[2] School of Civil & Environmental Engineering and School of Biological Sciences, Georgia Institute of Technology, Atlanta, Georgia, USA

[3] LABAQUA, S.A.U. Alicante, 03114, Spain

[4] TBI, Université de Toulouse, CNRS, INRAE, INSA, Toulouse, 31077, France

[5] Multidisciplinary Institute of Environmental Studies Ramon Margalef, University of Alicante, Alicante, 03080, Spain

[6] Applied Biomedicine Group (Alicante Institute for Health and Biomedical Research, ISABIAL), Alicante, 03080, Spain

Correspondence to: Fernando Santos

E-mail: Fernando.Santos@ua.es

Tel. +34 965 90 38 53

Table S1. Main features of the biofilm and water samples used.

Segment id	Sampling date	Water origin[a]	Water treatment[b]	Pipe material[c]
A	25.05.21	Mixed	CHLr	EPDM
B	25.05.21	Mixed	CHLr	EPDM
C	31.05.21	Mixed	CHLr	EPDM
D	7.06.21	Mixed	CHLr	EPDM
E	14.06.21	Mixed	CHLr	EPDM
F	21.06.21	Mixed	CHLr	EPDM
G	23.05.22	Mixed	CHLr	EPDM

a. Mixed waters include groundwaters with traces of coastal waters.

b. Water disinfection treatment in the DWDS: chlorination (CHLr)

c. Pipe material: ethylene propylene diene monomer (EPDM)

Table S2. List of the probes used to determine the distribution of bacterial phyla in biofilm samples with CARD-FISH.

Probe	Sequence (5' - 3')	Specificity	Formamide (%)/NaCl (mM)	Reference
ALF968	GGTAAGGTTCTGCGCGTT	*Alphaproteobacteria*	20/225	Neef,1997
BET42a	GCCTTCCCACTTCGTTT	*Betaproteobacteria*	35/80	Manz et al., 1992
CF319a	TGGTCCGTGTCTCAGTAC	*Bacteroidota*	35/80	Manz et al., 1996
CYA361	CCCATTGCGGAAAATTCC	*Cyanobacteria*	35/80	Schönhuber et al., 1999
EUB338-I	GCTGCCTCCCGTAGGAGT	Bacteria	35/80	Amann et al., 1990
EUB338-II	GCAGCCACCCGTAGGTGT	*Planctomycetota*	35/80	Daims et al., 1999
GAM42a	GCCTTCCCACATCGTTT	*Gammaproteobacteria*	35/80	Manz et al., 1992
HGC69a	TATAGTTACCACCGCCGT	*Actinomycetota*	25/159	Roller et al., 1994
NON338	ACTCCTACGGGAGGCAGC	Negative Control	0/900	Wallner et al., 1993

Table S3. Physicochemical parameters of the water samples analyzed in this study.

	Units	A	B	C	D	E	F	G
Conductivity 20ºC	µS/cm	883	988	980	1,075	961	1051	902
pH	U. pH.	8.4	8.3	8.3	8.4	8.3	8.2	8.2
Ammonia	mg/L	0.08	0.08	< 0.05	< 0.05	< 0.05	0.2	< 0.05
Sulphur	mg/L	46.39	52.11	49.24	46.84	51.62	51.06	32.73
Aluminum	µg/L	28	7	2	27	26	8	16
Barium	µg/L	26	29	28	31	26	29	18
Cobalt	µg/L	< 2	< 2	< 2	< 2	< 2	3	< 2
Copper	µg/L	7	< 2	59	< 2	< 2	2	< 2
Manganese	µg/L	< 2	< 2	< 2	< 2	< 2	75	< 2
Nickel	µg/L	< 2	52	22	< 2	< 2	303	< 2
Lead	µg/L	< 1	< 1	8	< 1	< 1	< 1	< 1
Selenium	µg/L	< 2	< 2	2	2	< 2	2	< 2
Zinc	µg/L	9	< 2	563	10	8	< 2	35
Total organic carbon (TOC)	mg/L	1	0.8	1	0.8	0.8	2.8	< 0.5
Total nitrogen	mg/L	1.9	2.3	2.3	2	1.7	3.4	1.4
Chloride	mg/L	149.3	179.8	179.8	225.5	177.2	201.4	172.4
Nitrate	mg/L	9.9	11.1	10.5	9.8	9	10.8	7
Sulfate	mg/L	160.3	169.6	165.1	165.3	168.7	174.4	99.5
Oxidability	mg O_2/L	0.7	0.57	0.7	0.83	0.76	1.91	0.75
Temperature	º C	25	24	25	25	25	24	22

Table S4. Counts of cells/ml and VLP/ml of water samples B, D and F. While different results were obtained in function of the technique used, the range of cells and virus-like particles (VLPs) per millimeter was around 10^4.

		B	D	F
**Cells/mL**	DAPI	1.44E+04	2.59E+04	3.33E+04
	Flow cytometry	2.55E+04	7.05E+04	1.35E+04
**VLPs/mL**	SYBR™ Gold	1.27E+05	5.00E+03	3.07E+04
	Flow cytometry	5.60E+04	2.55E+04	6.55E+04

Table S5. Taxonomic affiliation and abundances of the OTUs obtained from the 14 biofilms and waters 16S rRNA gene libraries. Samples have been normalized based on the sample with less sequences. OTUs present in ≥50% of water samples and ≥50% of biofilms, with no significant differences in their relative abundances between both types of samples, were considered as "generalist". Those OTUs present in biofilms and water samples, with a relative abundance statistically higher in one sample type were considered as "prevalent". OTUs exclusively present in ≥50% samples of a unique sample type were considered as "specific".

Table S6. Taxonomic affiliation and relative abundances (%) of the genera obtained from the 14 biofilms and waters 16S rRNA gene libraries. Genera present in ≥50% of water samples and ≥50% of biofilms, with no significant differences in their relative abundances between both types of samples, were considered as "generalist". Those genera present in biofilms and water samples, with a relative abundance statistically higher in one sample type were considered as "prevalent". Genera exclusively present in ≥50% samples of a unique sample type were considered as "specific".

Table S7. Metagenomes characteristics. N50 represents the length of the shortest contig in the smallest set of contigs that collectively contain at least 50% of the total assembly length.

	Reads						Assembly				
	Sample type	Total reads	Total base pair (bp)	Average length	% GC	Nonpareil coverage (diversity)	Contigs	bp assembled	% *reads* assembled	N50	Total ORFs
BM_A	Biofilm	1.75E+08	2.50E+10	150	64.286	98.28 (16.31)	8.07E+04	1.28E+08	98.87	5.49E+03	1.73E+05
BM_B	Biofilm	1.69E+08	2.35E+10	150	62.701	96.99 (18.14)	2.58E+05	3.77E+08	97.51	9.77E+03	5.19E+05
BM_C	Biofilm	1.72E+08	2.50E+10	150	63.809	97.97 (16.47)	1.37E+05	2.29E+08	98.67	1.90E+04	2.96E+05
BM_D	Biofilm	1.95E+08	2.59E+10	150	62.093	97.98 (16.73)	2.22E+05	3.09E+08	98.44	3.17E+03	3.94E+05
BM_E	Biofilm	1.80E+08	2.56E+10	150	63.248	97.74 (17.75)	2.23E+05	2.87E+08	98.20	7.88E+03	3.89E+05
BM_F	Biofilm	1.94E+08	2.70E+10	150	60.762	97.34 (17.84)	3.59E+05	4.58E+08	97.49	4.61E+03	6.63E+05
BM_G	Biofilm	4.40E+05	6.54E+07	151	64.711	92.07 (15.17)	4.07E+03	5.93E+07	90.52	6.27E+03	8.90E+03
WM_B	Water	5.34E+07	7.84E+09	151	59.706	99.02 (17.11)	1.08E+05	7.53E+09	95.64	3.81E+03	2.04E+05
WM_C	Water	5.23E+07	7.70E+09	151	64.483	98.84 (15.7)	5.74E+04	7.44E+09	96.35	7.33E+03	1.24E+05
WM_F	Water	5.10E+07	7.44E+09	151	61.36	97.7 (18.27)	2.05E+05	6.89E+09	92.05	4.97E+03	4.10E+05

Table S8. Taxonomic affiliation and relative abundances of the 16S rRNA genes reads extracted from the metagenomes.

Table S9. Functional annotation of contigs above 1kb.

Table S10. Genomospecies quality, taxonomy and relative abundances.

Table S11. Functional annotation of the genomospecies.

Table S12. Viral OTUs. Abundance, taxonomy and host association.
